# The association between dietary fat quality and quantity and hospitalization duration in COVID-19 in Iranian patients: a cross-sectional study

**DOI:** 10.3389/fnut.2025.1551760

**Published:** 2025-04-22

**Authors:** Farideh Shiraseb, Atieh Mirzababaei, Mahya Mehri Hajmir, Sara Ebrahimi, Shabnam Hosseini, Zeinab Zarrinvafa, Mahnaz Sadid, Yasaman Aali, Azam Mohamadi, Khadijeh Mirzaei

**Affiliations:** ^1^Department of Community Nutrition, School of Nutritional Science and Dietetics, Tehran University of Medical Sciences (TUMS), Tehran, Iran; ^2^Chronic Diseases Research Center, Endocrinology and Metabolism Population Sciences Institute, Tehran University of Medical Sciences, Tehran, Iran; ^3^Department of Exercise and Nutrition Sciences, Milken Institute School of Public Health, The George Washington University, Washington, DC, United States; ^4^School of Exercise and Nutrition Sciences, Institute for Physical Activity and Nutrition, Deakin University, Geelong, VIC, Australia; ^5^Faculty of Agricultural and Environmental Sciences, School of Human Nutrition, McGill University, Montreal, QC, Canada; ^6^Department of Nutrition, Science and Research Branch, Islamic Azad University, Tehran, Iran; ^7^Amir Alam Hospital, Internal Department, Tehran University Medical of Science, Tehran, Iran; ^8^Department of Nutrition, Faculty of Medicine, Mashhad University of Medical Sciences, Mashhad, Iran; ^9^Imam Khomeini Hospital Complex, Tehran University of Medical Sciences, Tehran, Iran

**Keywords:** fat quantity, fat quality, COVID-19, hospitalization duration, symptom

## Abstract

**Background:**

The global impact of Coronavirus Disease 2019 (COVID-19 (has highlighted the necessity of understanding factors influencing its severity and hospitalization duration. While a balanced diet is crucial for immune support, the role of dietary fats in this context has not been well understood. This study explored associations between the quality and quantity of fatty acids and severity and the length of hospitalization in COVID-19 patients in 2022.

**Method:**

This cross-sectional study included 107 COVID-19 patients aged 20–60 years who were hospitalized at Amir Alam Hospital in Tehran, Iran. Dietary fat intake was assessed using 24 h food recall. Data on symptoms were collected using a demographic questionnaire and verified against their hospital records. Linear and binary logistic regressions were employed for statistical analysis.

**Result:**

A higher omega 6/omega 3(N6/N3) ratio was linked to increased odds of respiratory distress syndrome (RDS) and elevated D-dimer levels, while correlating with lower odds of fever. While RDS odds increased over Vit E/polyunsaturated fatty acid (PUFA) ratio tertiles, chills decreased. [PUFA + monounsaturated fatty acid (MUFA)]/saturated fatty acid (SFA) ratio was associated with reduced odds of chest pain, duration of hospitalization (DH) time, c-reactive protein (CRP), and D-dimer levels. Furthermore, PUFA intake was negatively associated with odds of poor appetite, RDS, and headaches, whereas SFA intake was positively associated with odds of fever. Additionally, there was a positive correlation between cholesterol-saturated index (CSI) levels and DH time (*P* < 0.7).

**Conclusion:**

Our findings indicate that higher N6/N3 and VitE/PUFA ratios were associated with increased RDS and D-dimer levels, while the VitE/PUFA ratio was linked to reduced chills. Higher (PUFA + MUFA)/SFA ratios were associated with lower chest pain, DH, CRP, and D-dimer levels. While higher PUFA intake was related to reduced poor appetite, RDS, and headache, higher SFA intake was linked to increased fever. Additionally, there was a positive association between CSI levels and DH. Current findings indicate that the quality and balance of dietary fats may play a crucial role in modulating inflammatory responses and clinical outcomes.

## Introduction

The World Health Organization (WHO) has reported the global outbreak of Coronavirus Disease 2019 (COVID-19), with a death toll of 6,988,679, turning it into a significant global public health crisis (1). The COVID-19 symptoms range from mild to severe, including fever, cough, shortness of breath, loss of taste and smell, and fatigue (2). The COVID-19 induces a systemic inflammatory response that leads to a prothrombotic state and microangiopathy, primarily due to endothelial dysfunction, immune dysregulation, and heightened inflammation. These factors lay a key role in disease severity and complications. In severe cases, 14% of patients develop critical illness, with 5% requiring intensive care and hospitalization (3).

Existing evidence indicated that the severity and duration of COVID-19 are influenced by factors including age, gender, pre-existing hypertension or diabetes, and dietary patterns (4–6). The immune response to COVID-19 is modulated by modifiable factors including nutrition, physical activity, medication use, and sleep quality (7, 8). A well-balanced diet that meets energy needs and provides essential nutrients supports immune function and enhances immunometabolism (8). Higher intake of animal fat is linked to increased COVID-19 mortality, while higher protein consumption and lower fat or unsaturated fatty acid intake are associated with improved COVID-19 recovery (4, 9).

The role of dietary fats, including different types and quantities, remains underexplored. Fatty acids play a vital role in modulating immune responses and influencing infectious diseases (10). The current evidence shows that a high-fat diet can elevate proinflammatory cytokines and neutrophil levels, highlighting the importance of both the quality and quantity of dietary fat (11). Several indices are used to assess fat quality and its impact on health outcomes including the cholesteric saturation index (CSI), the omega 6/omega 3(N6/N3) ratio, and the [polyunsaturated fatty acid (PUFA) + monounsaturated fatty acid (MUFA)]/saturated fatty acid (SFA) ratio. The CSI predicts the effect of dietary fat composition on serum cholesterol levels with a lower CSI indicating a healthier fat profile (7). Moreover, the N6/N3 ratio plays a crucial role in inflammation regulation (7). The (PUFA + MUFA)/SFA ratio assesses the balance between unsaturated fats (PUFA and MUFA) and SFA. Replacing saturated fats with unsaturated fats has been shown to reduce low-density lipoprotein (LDL) cholesterol and improve cardiovascular health (12). A higher ratio, indicating excessive omega-6 intake relative to omega-3, is associated with increased inflammation (13). The VitE/PUFA ratio is crucial for preventing oxidative stress, as PUFAs are susceptible to lipid peroxidation, requiring adequate vitamin E to protect against cellular damage (10). While omega-3 PUFAs can reduce inflammatory markers, excessive saturated fat may activate immune pathways leading to inflammation (10, 14). These indices evaluate the quality of dietary fats and their potential health effects (15–20). While previous studies have explored the impact of dietary fats on immune responses, the evidence on their effects on COVID-19 severity is limited (21). This study aims to address this gap by investigating associations between the quality and quantity of dietary fats and the severity and duration of hospitalization in COVID-19 patients.

## Methods

### Study design and population

This cross-sectional study recruited patients with COVID-19 attending Amir Alam Hospital, in Tehran, Iran. A total of 107 adults (both male and female), aged 20–60 years, were recruited using a random sampling method between February 2020 and March 2020. The inclusion criteria were age between 20–60 years old, the presence of symptoms such as fever, chills, sore throat, sneezing, shortness of breath, chest pain, and other common symptoms. Data on symptoms were collected using a demographic questionnaire and verified against polymerase chain reaction (RT-PCR) testing of nasopharyngeal and oropharyngeal samples, along with biochemical evaluation that included CRP and D-dimer levels. All patients were diagnosed with COVID-19 according to WHO interim guidance, which mandates standard confirmation of acute SARS-CoV-2 infections based on the detection of unique viral sequences by nucleic acid amplification tests (NAATs), such as real-time reverse-transcription polymerase chain reaction (rRT-PCR). The inclusion criteria excluded individuals with kidney or liver abnormalities. Furthermore, patients with rare viral diseases such as Human Immunodeficiency Virus (HIV), or those who had undergone chemotherapy in the past month, were not included in the study. The discharge criteria were the absence of fever for at least 3 days, substantial improvement in both lungs on chest computed tomography (CT), clinical remission of respiratory symptoms, and two negative throat-swab samples obtained at least 24 h apart.

The research protocol was approved by the research committee and all the signed Patients a written consent form (IR.TUMS.MEDICINE.REC.1399.433).

### Demographic and anthropometric assessment

Baseline characteristics of patients, including their age, gender, marital status, education level, occupation, family history of obesity, and socioeconomic status, were collected using structured and closed-ended questionnaires through interviews conducted by a trained nutritionist. Due to the disease conditions and the inability to approach patients directly. A Seca digital scale (Germany) with a precision of 0.1 kg was used to assess participants' body weight. Height was measured using a Seca 206 stadiometer (Germany) with a precision of 0.1 cm.

### Dietary assessment

Three non-consecutive 24 h dietary recalls were used to collect food and beverage intake over 2-week days and one weekend. It was ensured that 24 h dietary recalls did not reflect hospital meals (11). The recalls were collected through interviews with patient's companion or family member, focusing on the patients' dietary intake prior to hospitalization. This approach was used to reflect habitual dietary patterns before the acute event that led to hospitalization.

### Fat quality and quantity assessment

Dietary fat intake was assessed using 24 h dietary recalls, with a focus on identifying specific dietary fat sources. The collected data were analyzed using N4 software, enabling precise quantification of various fatty acids, including PUFA (g/day), MUFA (g/day), SFA (g/day), omega-6 (g/day), omega-3 (g/day), and vitamin E (mg/day). To evaluate dietary fat quality, several indices and ratios were calculated including the (PUFA + MUFA)/SFA ratio, CSI, N6/N3, and the VitE/PUFA ratio, commonly used measured of the health implications of dietary fat composition. Caloric intake was controlled, ranging from 800 to 4,500 kcal per day with all patient's intake levels falling within this range to ensure consistency in the dietary assessment.

### COVID-19 symptoms assessment

Patients were asked to complete a general questionnaire to obtain information on the presence of common clinical symptoms of COVID-19 (taste, smell, appetite, lethargy, chest pain, headache, chills, fever, cough, confusion, sore throat, nausea, RDS, and vomiting). These symptoms have been confirmed by a physician.

### Biochemical sign

Biochemical evaluation including CRP (mg/L) and D-dimer (ng/ml) was collected. Twelve milliliters of blood were collected from each patient, who were fasted for 10 to 12 h overnight. The blood was transferred into two tubes: one was containing Ethylenediaminetetraacetic Acid **(**EDTA) as an anticoagulatory factor and one without it. Half of the tube without EDTA was immediately centrifuged for 15 min and after removing the serum, it was stored at −80°C.

### Assessment of other variables

The International Physical Activity Questionnaire (IPAQ) was used to assess physical activity, duration of convalescence, supplements intake, and the use of corticosteroids or antiviral medications for each Patient.

### Statistical analysis

Data analysis was performed using SPSS software version 26. Given the challenges of data collection during the COVID-19 pandemic, this method was selected to optimize the use of available data. Furthermore, a *post-hoc* power calculation was performed to evaluate the adequacy of the sample size, confirming that the study maintained sufficient statistical power. The power of sample size was determined using the event per variable (EPV) approach, ensuring a minimum of 10 events per predictor variable to maintain statistical power. The data were analyzed using SPSS software version 21. The *P-*value (*P* < 0.05) was considered statistically significant. *P-values* between 0.05 and 0.07 were considered marginally significant. The normality of continuous variables was assessed using the Kolmogorov-Smirnov test (*P* > 0.05). The mean and standard deviation (SD) were reported for continuous variables, while numbers and percentages were reported for categorical variables. One-way analysis of variance (ANOVA) and Chi-square tests (χ^2^) were used to compare continuous and categorical variables, respectively. Analysis of covariance (ANCOVA) was used for the analysis adjusted for confounders. Linear Regression analysis was conducted for continuous outcomes, with results reported by beta (β) and 95% confidence interval (CI). Binary logistic regression analysis has been performed for binary outcomes, reporting odds ratio (OR) and 95% CI. Model 1 was adjusted for age, sex, education level, body mass index (BMI), and physical activity. Model 2 was adjusted further with comorbidity, medication use, and supplementation intake. For binary logistic regression, the following variables were considered the reference group: absence of chest pain, headache, nose, vomiting, nausea, diarrhea, sore throat, stomach pain, joint pain, confusion, contusion, chills, and respiratory distress syndrome (RDS). *P-*value < 0.05 was considered statistically significant, while *P-*values between 0.05 and 0.07 were considered marginally significant.

## Result

### Characteristics of the study patients

This study included 107 patients. The characteristics of participants are presented in Supplementary Table 1. The mean age and mean BMI were 46.21 years, and 29.36 kg/m^2^, respectively. The majority of participants were males (66%), had a university-level education (53%) and were engaged in self-employment (58%).

### Symptoms and duration of hospitalization across tertiles of dietary fat quality indices

Symptoms and hospitalization duration over tertiles of dietary fat quality indices were shown in Supplementary Table 2. After controlling for confounders CRP levels (*P-*value = 0.03) were significantly lower while confusion (*P-*value = 0.02) was higher over tertiles of (PUFA +MUFA)/ SFA. Furthermore, participants showed fewer chill symptoms over tertiles of the VitE/PUFA (*P-*value = 0.03). In the adjusted model, the mean level of D-dimer was significantly higher (*P-*value = 0.03), while the level of CRP was marginally lower across the tertiles of N6/N3 (*P-*value = 0.07). Furthermore, vomiting was significantly higher over the top tertile of CSI (*P-*value = 0.07).

### Symptoms and hospitalization duration across the tertiles of dietary fat quantity

Symptoms and duration of hospitalization across the tertiles of dietary fat quantity are presented in Supplementary Table 3. In the adjusted model, individuals with linolenic acid intake exceeding 0.73 g per day had a significantly higher mean of hospitalization day (*P-*value = 0.01), and appetite (*P-*value = 0.06) while had a marginally significant lower levels of lethargy (*P-*value = 0.07). After adjusting for confounders, participants with higher intake of oleic acid (≥33.68 g/d) had lower recovery time (*P-*value = 0.07) and D-dimer levels (*P-*value = 0.05), while had higher mean level of chills (*P-*value = 0.06). In the adjusted model, the mean level of chills was significantly higher with a higher intake of MUFA (≥35.89 g/d) (*P-*value = 0.04). In the adjusted model, the mean levels of D-dimer were significantly higher with increased intake of cholesterol (≥183.6 mg/d) (*P-*value = 0.04).

### Associations between quality of fatty acids and symptoms of COVID-19

The relationship between the quality of fatty acids and the symptoms of COVID-19 is outlined in Table 1. There were significantly higher odds of respiratory distress syndrome (RDS) over N6/N3 ratio tertiles in model 2 (OR = 4.40, 95% CI = 1.18, 16.28; *P-*value = 0.02), compared to tertile 1 (*P*-trend = 0.02). Additionally, significantly lower odds of fever were associated with the higher N6/N3 ratio (OR = 0.26, 95% CI = 0.07, 0.92; *P-*value = 0.03) (*P*-trend = 0.03). Furthermore, a higher intake of N6/N3 was associated with increased odds of appetite compared to tertile 1 (OR = 3.62, 95% CI = 0.89, 14.59; *P-*value = 0.07).

**Table 1:** The association of quality of fatty acids with sign and symptoms of COVID-19 (*N* = 107).

**Variables**	**T2 (*n* = 35)**	***P-*value**	**T3 (*n* = 35)**	***P-*value**	***P*-trend**	**T2 (*n* = 35)**	***P-*value**	**T3 (*n* = 35)**	***P-*value**	***P*-trend**
**OR (95%CI)**										
	**N6/N3**					**CSI**				
**Taste**										
Crude	1.55 (0.26, 8.98)	0.62	1.18 (0.21, 6.66)	0.85	0.85	1.49 (0.63, 8.47)	0.65	2.21 (0.39, 12.41)	0.36	0.36
Model 1	1.26 (0.21, 7.61)	0.79	0.94 (0.16, 5.50)	0.95	0.95	1.55 (0.27, 8.35)	0.62	2.34 (0.39, 13.58)	0.34	0.34
Model 2	0.91 (0.15, 5.39)	0.92	0.86 (0.15, 4.80)	0.86	0.86	1.75 (0.32, 9.56)	0.51	2.09 (0.37, 11.72)	0.40	0.39
**Smell**										
Crude	0.55 (0.09, 3.23)	0.51	0.76 (0.13, 4.34)	0.75	0.76	2.54 (0.44, 14.48)	0.29	0.64 (0.11, 3.59)	0.64	0.62
Model 1	0.59 (0.09.3.65)	0.57	0.76 (0.13, 4.54)	0.76	0.76	2.36 (0.41, 13.41)	0.33	0.49 (0.08, 2.88)	0.43	0.44
Model 2	0.62 (0.10, 3.88)	0.61	0.66 (0.11, 3.38)	0.64	0.64	2.49 (0.44, 13.91)	0.29	0.48 (0.08, 2.77)	0.41	0.42
**Appetite**										
Crude	0.67 (0.16, 2.78)	0.58	2.61 (0.64, 10.66)	0.18	0.18	0.92 (0.21, 3.89)	0.91	0.96 (0.23, 4.01)	0.95	0.95
Model 1	0.63 (0.15, 2.69)	0.54	3.51 (0.85, 14.45)	0.08	0.08	0.96 (0.22, 4.07)	0.95	1.00 (0.23, 4.36)	0.99	0.99
Model 2	0.56 (0.13, 2.39)	0.43	3.62 (0.89, 14.59)	**0.07**	0.80	0.95 (0.22, 3.95)	0.94	0.92 (0.21, 3.95)	0.91	0.91
**Lethargy**										
Crude	1.45 (0.38, 5.48)	0.57	2.44 (0.66, 9.02)	0.18	0.18	1.52 (0.40, 5.72)	0.53	0.80 (0.21, 2.97)	0.73	0.74
Model 1	1.41 (0.36, 5.51)	0.61	2.46 (0.64, 9.33)	0.18	0.18	1.59 (0.42, 6.04)	0.48	0.95 (0.24, 3.70)	0.94	0.95
Model 2	1.82 (0.46, 7.09)	0.38	2.57 (0.69, 9.60)	0.15	0.15	1.44 (0.39, 5.38)	0.58	0.97 (0.25, 3.71)	0.97	0.97
**RDS**										
Crude	0.28 (0.35, 4.68)	0.70	3.00 (0.92, 9.69)	0.06	**0.05**	0.90 (0.28, 2.84)	0.86	1.03 (0.34, 3.15)	0.94	0.95
Model 1	1.45 (0.36, 5.81)	0.59	3.89 (1.09, 13.81)	**0.03**	**0.03**	0.78 (0.23, 2.59)	0.68	0.87 (0.26, 2.85)	0.82	0.82
Model 2	1.35 (0.33, 5.54)	0.67	4.40 (1.18, 16.28)	**0.02**	**0.02**	0.78 (0.23, 2.63)	0.70	0.86 (0.26, 2.85)	0.81	0.81
**Fever**										
Crude	0.40 (0.12, 1.32)	0.13	0.26 (0.08, 0.83)	**0.02**	**0.02**	1.05 (0.38, 2.93)	0.91	1.48 (0.51, 4.29)	0.46	0.47
Model 1	0.41 (0.11, 1.44)	0.16	0.26 (0.07, 0.87)	**0.02**	**0.02**	1.05 (0.36, 3.01)	0.92	1.35 (0.45, 4.22)	0.56	0.57
Model 2	0.46 (0.12, 1.69)	0.24	0.26 (0.07, 0.92)	**0.03**	**0.03**	0.94 (0.31, 2.80)	0.92	1.46 (0.48, 4.60)	0.51	0.52
**Chills**										
Crude	0.84 (0.31, 2.25)	0.73	0.73 (0.27, 1.91)	0.52	0.52	0.91 (0.34, 2.40)	0.85	0.84 (0.32, 2.21)	0.73	0.73
Model 1	0.93 (0.32, 2.64)	0.89	0.65 (0.23, 1.82)	0.41	0.41	0.82 (0.29, 2.27)	0.70	0.72 (0.25, 2.00)	0.53	0.53
Model 2	1.03 (0.35, 3.02)	0.94	0.68 (0.24, 1.92)	0.46	0.47	0.77 (0.27, 2.17)	0.63	0.72 (0.25, 2.04)	0.54	0.54
Crude	0.75 (0.29, 1.93)	0.55	1.05 (0.40, 2.69)	0.91	0.91	0.63 (0.24, 1.63)	0.34	1.07 (0.41, 2.76)	0.88	0.88
Model 1	0.72 (0.26, 1.92)	0.52	0.95 (0.35, 2.55)	0.92	0.91	0.56 (0.21, 1.51)	0.26	0.91 (0.33, 2.49)	0.86	0.87
Model 2	0.71 (0.25, 2.00)	0.52	1.00 (0.36, 2.73)	0.99	0.99	0.55 (0.20, 1.50)	0.24	0.96 (0.35, 2.65)	0.94	0.94
**Confusion**										
Crude	0.49 (0.16, 1.47)	0.20	1.08 (0.40, 2.87)	0.87	0.85	0.72 (0.25, 2.07)	0.53	0.91 (0.33, 2.47)	0.86	0.86
Model 1	0.45 (0.14, 1.43)	0.17	1.19 (0.41, 3.39)	0.74	0.76	0.81 (0.26, 2.56)	0.73	1.15 (0.37, 3.52)	0.80	0.78
Model 2	0.43 (0.13, 1.40)	0.16	1.15 (0.40, 3.32)	0.79	0.82	0.84 (0.26, 2.70)	0.78	1.20 (0.38, 3.73)	0.74	0.72
**Sore throat**										
Crude	1.04 (0.37, 2.87)	0.93	1.09 (0.40, 2.95)	0.86	0.86	1.09 (0.40, 2.92)	0.86	080 (0.29, 2.19)	0.66	0.67
Model 1	0.84 (0.28, 2.51)	0.76	1.33 (0.46, 3.85)	0.59	0.60	1.73 (0.56, 5.36)	0.33	1.06 (0.34, 3.29)	0.91	0.87
Model 2	0.82 (0.26, 2.61)	0.75	1.29 (0.43, 3.84)	0.64	0.65	1.92 (0.60, 6.14)	0.26	1.21 (0.37, 3.93)	0.74	0.77
**Nausea**										
Crude	0.83 (0.32, 2.15)	0.70	0.84 (0.33, 2.16)	0.73	0.73	1.10 (0.42, 2.86)	0.85	1.48 (0.57, 3.80)	0.41	0.41
Model 1	1.05 (0.38, 2.93)	0.91	0.82 (0.30, 2.25)	0.70	0.71	0.82 (0.28, 2.42)	0.73	1.04 (0.36, 2.98)	0.93	0.91
Model 2	1.08 (0.37, 3.10)	0.88	0.89 (0.31, 2.51)	0.83	0.84	0.78 (0.25, 2.34)	0.65	1.04 (0.35, 3.07)	0.93	0.90
**Vomiting**										
Crude	0.46 (0.10, 2.04)	0.31	0.78 (0.21, 2.83)	0.70	0.70	0.77 (0.16, 3.74)	0.53	2.00 (0.52, 7.55)	0.30	0.27
Model 1	0.50 (0.15, 2.43)	0.39	0.64 (0.15, 2.68)	0.55	0.54	0.60 (0.10, 3.71)	0.59	1.92 (0.41, 8.092)	0.40	0.30
Model 2	0.49 (0.09, 2.50)	0.39	0.67 (0.15, 2.89)	0.59	0.55	0.46 (0.07, 3.01)	0.42	1.68 (0.35, 8.14)	0.50	0.49
**Headache**										
Crude	1.06 (0.41, 2.73)	0.90	0.94 (0.37, 2.39)	0.90	0.90	0.44 (0.17, 1.15)	0.09	0.95 (0.37, 2.44)	0.91	0.90
Model 1	1.37 (0.50, 3.76)	0.53	1.05 (0.38, 2.85)	0.92	0.91	0.27 (0.09, 0.83)	**0.02**	0.58 (0.20, 1.70)	0.32	0.33
Model 2	1.40 (0.49, 3.97)	0.52	1.13 (0.40, 3.11)	0.81	0.80	0.26 (0.08, 0.80)	**0.02**	0.58 (0.19, 1.74)	0.33	0.44
**Chest pain**										
Crude	0.75 (0.29, 1.93)	0.55	0.67 (0.26, 1.71)	0.40	0.40	0.50 (0.19, 1.29)	0.15	0.84 (0.33, 2.16)	0.73	0.72
Model 1	0.81 (0.29, 2.22)	0.68	0.64 (0.23, 1.72)	0.37	0.38	0.33 (0.11, 0.98)	**0.04**	0.62 (0.21, 1.80)	0.38	0.44
Model 2	0.82 (0.29, 2.32)	0.72	0.67 (0.24, 1.82)	0.43	0.43	0.30 (0.10, 0.93)	**0.03**	0.60 (0.20, 1.76)	0.35	0.43
Crude	1.57 (0.28, 8.86)	0.43	0.43 (0.07, 2.46)	0.34	0.34	1.55 (0.26, 8.98)	0.62	1.18 (0.26, 6.66)	0.85	0.85
Model 1	1.84 (0.32, 10.47)	0.49	0.46 (0.80, 2.67)	0.38	0.34	1.26 (0.21, 7.61)	0.79	0.94 (0.16, 5.50)	0.95	0.95
Model 2	1.07 (0.18, 6.15)	0.93	0.35 (0.06, 1.97)	0.23	0.37	0.94 (0.15, 5.39)	0.92	0.86 (0.15, 4.80)	0.86	0.86
**Smell**										
Crude	0.32 (0.05, 1.83)	0.20	0.25 (0.04, 1.48)	0.12	0.13	0.62 (0.10, 3.88)	0.61	0.66 (0.11, 3.86)	0.64	0.64
Model 1	0.32 (0.05, 1.87)	0.20	0.26 (0.04, 1.56)	0.14	0.13	0.59 (0.09, 3.65)	0.57	0.76 (0.13, 4.51)	0.76	0.76
Model 2	0.28 (0.04, 1.70)	0.25	0.28 (0.04, 1.46)	0.12	0.22	0.55 (0.09, 3.23)	0.51	0.76 (0.13, 4.34)	0.75	0.76
**Appetite**										
Crude	2.00 (0.48, 8.34)	0.33	2.96 (0.70, 12.44)	0.13	0.13	0.67 (0.16, 2.78)	0.58	2.61 (0.64, 10.66)	0.18	0.18
Model 1	2.07 (0.49, 8.71)	0.32	3.01 (0.70, 12.85)	0.13	0.13	0.63 (0.15, 2.69)	0.54	3.51 (0.85, 14.45)	0.08	0.08
Model 2	1.78 (0.41, 7.7)	0.43	2.80 (0.66, 11.86)	0.16	0.16	0.56 (0.13, 2.39)	0.43	3.62 (0.89, 14.59)	0.07	0.08
**Lethargy**										
Crude	0.51 (0.13, 1.92)	0.32	1.04 (0.27, 3.90)	0.94	0.42	0.52 (0.13, 1.92)	0.31	1.06 (0.27, 3.89)	0.38	0.38
Model 1	0.61 (0.15, 2.34)	0.47	1.22 (0.29, 5.03)	0.78	0.71	0.60 (0.15, 2.34)	0.45	1.24 (0.29, 5.02)	0.70	0.70
Model 2	0.81 (0.20, 0.32)	0.76	1.45 (0.35, 5.09)	0.60	0.58	0.80 (0.20, 0.32)	0.74	1.47 (0.35, 5.08)	0.57	0.57
**RDS**										
Crude	4.54 (1.14, 18.09)	**0.03**	3.57 (0.87, 14.60)	0.07	0.09	0.77 (0.23, 2.58)	0.44	1.54 (0.50, 4.67)	0.67	0.08
Model 1	5.43 (1.24, 23.76)	**0.02**	4.51 (0.96, 21.19)	**0.05**	0.07	0.68 (0.19, 2.46)	0.56	1.68 (0.50, 5.62)	0.39	0.38
Model 2	5.73 (1.24, 26.47)	**0.02**	4.54 (0.95, 21.74)	**0.05**	**0.04**	0.68 (0.18, 2.56)	0.57	1.79 (0.51, 6.18)	0.35	0.34
**Fever**										
Crude	0.16 (0.04, 0.57)	**< 0.01**	0.38 (0.10, 0.57)	0.14	0.70	0.34 (0.10, 1.11)	0.34	0.33 (0.10, 1.07)	0.33	0.60
Model 1	0.12 (0.03, 0.46)	**< 0.01**	0.30 (0.07, 1.24)	0.09	0.10	0.34 (0.10, 1.43)	0.08	0.32 (0.09, 1.18)	0.08	0.08
Model 2	0.13 (0.03, 0.52)	**< 0.01**	0.30 (0.07, 1.30)	0.10	0.13	0.25 (0.06, 0.94)	**0.04**	0.24 (0.06, 0.91)	**0.03**	0.43
**Chills**										
Crude	0.21 (0.07, 0.64)	**< 0.01**	0.25 (0.08, 0.76)	**< 0.01**	**< 0.01**	0.54 (0.20, 1.42)	0.21	1.36 (0.49, 3.76)	0.55	0.55
Model 1	0.13 (0.03, 0.46)	**< 0.01**	0.16 (0.04, 0.60)	**< 0.01**	**< 0.01**	0.59 (0.22, 1.62)	0.31	1.28 (0.42, 3.85)	0.65	0.69
Model 2	0.12 (0.03, 0.48)	**< 0.01**	0.15 (0.04, 0.60)	**< 0.01**	**0.01**	0.35 (0.19, 1.49)	0.23	1.18 (0.38, 3.62)	0.76	0.79
Crude	3.77 (1.39, 10.21)	**< 0.01**	1.80 (0.66, 4.87)	0.24	0.93	0.75 (0.29, 1.93)	0.55	1.05 (0.40, 2.69)	0.91	0.91
Model 1	0.23 (0.08, 0.67)	**< 0.01**	0.52 (0.17, 1.58)	0.25	0.29	0.72 (0.26, 1.95)	0.52	0.95 (0.35, 2.55)	0.92	0.97
Model 2	0.23 (0.07, 0.70)	0.01	0.51 (0.16, 1.57)	0.24	0.28	0.71 (0.25, 1.00)	0.52	1.00 (0.36, 2.73)	0.99	0.99
**Confusion**										
Crude	1.76 (0.64, 4.87)	0.27	0.96 (0.32, 2.87)	0.94	0.97	0.08 (−1.01, 1.17)	0.88	0.89 (−0.14, 1.92)	0.09	0.08
Model 1	0.169 (0.57, 4.97)	0.33	0.80 (0.24, 2.64)	0.71	0.70	−0.02 (−1.17, 1.12)	0.96	0.59 (−0.50, 1.70)	0.29	0.27
Model 2	1.77 (0.58, 544)	0.31	0.79 (0.23, 2.68)	0.71	0.68	0.01 (−1.14, 1.69)	0.98	0.61 (−0.50, 1.73)	0.27	0.26
**Sore throat**										
Crude	0.92 (0.32, 2.60)	0.88	1.60 (0.58, 4.35)	0.35	0.32	1.76 (0.64, 4.87)	0.27	1.27 (0.44, 3.61)	0.65	0.61
Model 1	0.82 (0.27, 2.47)	0.72	1.21 (0.40, 3.73)	0.72	0.71	1.76 (0.60, 5.14)	0.30	1.71 (0.54, 5.42)	0.35	0.34
Model 2	0.94 (0.28, 3.11)	0.92	1.31 (0.39, 4.33)	0.65	0.62	2.08 (0.67, 6.43)	0.20	1.76 (0.54, 5.70)	0.34	0.74
**Nausea**										
Crude	0.34 (0.13, 0.92)	**< 0.01**	0.59 (0.22, 1.53)	0.28	0.28	0.50 (0.19, 1.31)	0.15	0.84 (0.32, 2.16)	0.72	0.72
Model 1	0.25 (0.08, 0.76)	**0.01**	0.53 (0.17, 1.60)	0.26	0.28	0.53 (0.19, 1.48)	0.23	0.85 (0.30, 2.42)	0.77	0.74
Model 2	0.24 (0.07, 0.75)	**0.01**	0.51 (0.16, 1.60)	0.25	0.28	0.47 (0.16, 1.36)	0.16	0.80 (0.27, 2.32)	0.68	0.78
**Vomiting**										
Crude	0.40 (0.11, 1.50)	0.17	0.19 (0.03, 1.00)	**0.05**	**< 0.01**	0.34 (0.06, 1.89)	0.21	1.45 (0.41, 5.11)	0.56	0.50
Model 1	0.27 (0.06, 1.17)	0.08	0.27 (0.01, 0.77)	**0.02**	**0.01**	0.32 (0.05, 1.95)	0.21	0.91 (0.23, 3.64)	0.90	0.88
Model 2	0.26 (0.05, 1.22)	0.09	0.13 (0.02, 0.86)	**0.03**	0.35	0.27 (0.04, 1.73)	0.16	0.79 (0.18, 3.38)	0.75	0.85
**Headache**										
Crude	1.11 (0.43, 2.85)	0.81	1.05 (0.41, 2.72)	0.90	0.92	0.34 (0.12, 0.98)	**0.03**	0.40 (0.15, 1.07)	0.06	0.07
Model 1	1.00 (0.37, 2.72)	0.99	1.12 (0.38, 3.28)	0.82	0.62	0.36 (.13, 1.02)	**0.05**	0.44 (0.15, 1.29)	0.13	0.13
Model 2	1.06 (0.37, 2.99)	0.91	1.14 (0.38, 3.40)	0.80	0.80	0.34 (0.11, 0.98)	**0.04**	0.42 (0.14, 1.25)	0.11	0.06
**Chest pain**										
Crude	1.57 (0.61, 4.05)	0.34	1.06 (0.41, 2.73)	0.90	0.89	0.34 (0.12, 0.98)	**0.03**	0.40 (0.15, 1.07)	**0.06**	**0.07**
Model 1	1.58 (0.58, 4.31)	0.36	1.18 (0.41, 3.36)	0.36	0.76	0.35 (0.12, 0.99)	**0.04**	0.35 (0.12, 1.03)	**0.05**	**0.05**
Model 2	1.70 (0.60, 4.82)	0.31	1.22 (0.42, 3.60)	0.70	0.73	0.32 (0.11, 0.95)	**0.03**	0.32 (0.10, 0.98)	**0.04**	**0.06**

DH, Duration of hospitalization; RDS, Respiratory distress syndrome; CRP, C-reactive protein; OR: odds ratio. Data were obtained from the binary regression. Model 1 was adjusted for age, sex, education level, BMI, and physical activity. Model 2 was adjusted age, sex, education level, BMI, physical activity, comorbidity, use of medication, and supplementation use. Data are presented as OR and 95% confidence interval. Model 1 adjusted for significant associations with a P-value < 0.05 are bolded.

Higher levels of CSI were associated with lower odds of headaches when comparing tertile 2 to tertile 1 (OR = 0.26; 95% CI = 0.08, 0.80; *P-*value = 0.02). Similarly, [patients in tertile 2 of adherence to CSI displayed lower odds of chest pain, compared to the first tertile (OR = 0.30; 95% CI = 0.10, 0.93; *P-*value = 0.03).

There were higher odds of RDS in the second (OR = 5.73; 95% CI = 1.24, 26.47; *P-*value = 0.02) and third (OR = 4.54; 95% CI: 0.95, 21.74; *P-*value = 0.05) tertiles of vitamin E/PUFA ratio (*P*-trend = 0.04). Furthermore, the second tertile of the vitamin E/PUFA ratio was associated with lower odds of fever compared to the first tertile (OR = 0.13; 95% CI = 0.03, 0.52; *P-*value < 0.01). Additionally, lower odds of chills were found in the second (OR = 0.12; 95% CI = 0.03, 0.48; *P-*value < 0.01) and third (OR = 0.15; 95% CI = 0.04, 0.60; *P-*value < 0.01) (*P*-trend = 0.01) tertiles of the Vitamin E/PUFA ratio. Lower odds of contusion (OR = 0.23; 95% CI = 0.07, 0.70; *P-*value = 0.01), and nausea (OR = 0.24; 95% CI = 0.07, 0.75; *P-*value = 0.01) were observed in the second tertile of the vitamin E/PUFA ratio. A lower odd of vomiting was found in the third tertile of the vitamin E/PUFA ratio (OR = 0.13, 95% CI = 0.02, 0.86; *P-*value = 0.03).

There were lower odds of fever in the second (OR = 0.25, 95% CI = 0.06, 0.94; *P-*value = 0.04), and third tertile (OR = 0.24, 95% CI = 0.06, 0.91; *P-*value = 0.03) of (PUFA +MUFA)/ SFA ratio compared to first tertile. Furthermore, lower odds of headache were observed in the second tertiles of (PUFA + MUFA)/SFA (OR = 0.34, 95% CI = 0.11, 0.98; *P-*value = 0.04) and lower odds of chest pain were observed in second (OR = 0.32, 95% CI = 0.11, 0.95; *P-*value = 0.03), and third (OR = 0.32, 95% CI = 0.10, 0.98; *P-*value = 0.04) (*P*-trend = 0.06).

### Associations between quality of fatty acids and symptom and hospitalization duration of COVID-19

Associations between fatty acid quality and COVID-19 symptom duration and hospitalization duration were presented in Table 2. Significant positive association was found between CSI levels and duration of hospitalization (DH) time (β = 1.39; CI = −0.03, 2.82; *P-*value = 0.05) (*P*-trend = 0.05).

**Table 2:** Association of quality of fatty acids with sign and duration of hospitalization of COVID-19 (*N* = 107).

**Variables**	**T2 (*n* = 35)**	***P-*value**	**T3 (*n* = 35)**	***P-*value**	***P*-trend**	**T2 (*n* = 35)**	***P-*value**	**T3 (*n* = 35)**	***P-*value**	***P*-trend**
β **(95%CI)**										
	**N6/N3**					**CSI**				
**Hospital stay duration (day)**										
Crude	−1.26 (−3.60, 1.70)	0.29	−1.22 (−3.53, 1.08)	0.29	0.30	0.53 (−1.79, 2.87)	0.65	−0.16 (−2.48, 2.15)	0.88	0.89
Model 1	−0.12 (−2.35, 2.10)	0.91	−1.07 (−3.26, 1.11)	0.33	0.33	0.73 (−1.59, 3.06)	0.53	0.16 (−2.20, 2.52)	0.89	0.89
Model 2	0.11 (−2.05, 2.28)	0.91	−1.27 (−3.37, 0.82)	0.23	0.23	0.71 (−1.56, 2.98)	0.54	0.05 (−2.25, 2.36)	0.96	0.96
**DH (day)**										
Crude	0.24 (−1.17, 1.67)	0.73	−0.78 (−2.19, 0.61)	0.27	0.27	1.08 (−0.32, 2.48)	0.13	1.35 (−0.06, 2.71)	0.06	0.06
Model 1	0.44 (−1.02, 1.91)	0.55	−0.76 (−2.20, 0.67)	0.29	0.30	1.17 (−0.24, 2.59)	0.10	1.46 (0.02, 2.91)	**0.04**	**0.04**
Model 2	0.49 (−0.97, 1.96)	0.50	−0.78 (−2.20, 0.63)	0.28	0.29	1.14 (−0.25, 2.54)	0.11	1.39 (−0.03, 2.82)	**0.05**	**0.05**
**CRP (mg/L)**										
Crude	71.67 (−14.58, 267.94)	0.47	83.72 (−109.75, 277.20)	0.39	0.39	40.194 (−223.4, 153.02)	0.68	155.29 (−347.0, 36.50)	0.11	**0.05**
Model 1	31.72 (−162.98, 226.43)	0.74	60.85 (−129.98, 251.69)	0.53	0.53	20.47 (−220.6, 179.64)	0.84	142.21 (−341.5, 57.14)	0.16	0.15
Model 2	65.40 (−128.93, 259.74)	0.50	95.86 (−135.43, 240.34)	0.58	0.57	27.54 (−225.10, 170.01)	0.78	138.11 (−334.47, 58.23)	0.16	0.15
**D-dimer (ng/ml)**										
Crude	1.18 (−6.64, 9.01)	0.76	5.35 (−3.34, 14.05)	0.22	0.24	229.54 (3.64, 445.62)	**0.04**	124.73 (−99.67, 349.14)	0.27	0.27
Model 1	3.94 (−3.27, 11.16)	0.28	12.18 (3.72, 20.64)	**< 0.01**	**< 0.01**	125.33 (−11.19, 361.85)	0.29	25.01 (−210.6, 260.61)	0.83	0.89
Model 2	2.55 (−4.62, 9.72)	0.48	12.61 (4.27, 20.96)	**< 0.01**	**< 0.01**	118.85 (−118.29, 356.01)	0.32	17.90 (−217.80, 253.60)	0.82	0.93
	**VitE/PUFA**					**(PUFA** +**MUFA)/SFA**				
**Recovery time (day)**										
Crude	0.90 (−1.41, 3.21)	0.44	−0.86 (−3.19, 1.45)	0.46	0.46	1.44 (−0.88, 3.76)	0.22	0.61 (−1.72, 2.95)	0.60	0.61
Model 1	1.15 (−1.04, 3.35)	0.30	0.15 (−2.15, 2.46)	0.89	0.90	1.64 (−0.53, 3.81)	0.14	0.50 (−1.77, 2.78)	0.66	0.64
Model 2	1.13 (−1.04, 3.31)	0.30	0.27 (−1.96, 2.51)	0.84	0.80	1.34 (−0.78, 3.47)	0.21	0.31 (−1.89, 2.51)	0.78	0.77
**DH (day)**										
Crude	−0.01 (−1.44, 1.41)	0.98	−0.15 (−1.58, 1.29)	0.83	0.83	0.37 (−1.04, 1.79)	0.60	−0.63 (−2.06, 0.79)	0.38	0.38
Model 1	−0.09 (−1.56, 1.37)	**< 0.01**	−0.16 (−1.71, 1.37)	**< 0.01**	0.83	0.41 (−1.03, 1.85)	0.57	−0.79 (−2.30, 0.71)	0.30	0.31
Model 2	−0.20 (−1.69, 1.28)	0.78	−0.17 (−1.70, 1.35)	0.81	0.82	0.25 (−1.18, 1.69)	0.73	−0.85 (−2.35, 0.63)	0.26	**0.06**
Crude	−132.27 (−325.95, 61.40)	0.18	−122.02 (−317.04, 72.99)	0.22	0.21	143.77 (−48.61, 336.16)	0.14	−29.64 (−223.3, 164.07)	0.76	0.75
Model 1	−103.76 (−294.54, 87.01)	0.28	−125.58 (−326.24, 75.07)	0.22	0.22	116.26 (−73.18, 305)	0.87	−15.73 (−214.10, 182.63)	0.22	**0.05**
Model 2	−72.49 (−266.23)	0.46	−106.89 (−305.9, 92.11)	0.29	0.29	110.86 (−77.48, 229.21)	0.24	−30.84 (−226.93, 165.25)	0.75	**0.06**
**D–dimer (ng/ml)**										
Crude	−113.93 (−343.1, 115.33)	0.33	−44.11 (−274.96, 186.74)	0.70	0.71	21.91 (−207.01, 250.85)	0.85	−101.46 (−331.98, 129.04)	0.38	0.38
Model 1	−68.90 (−29.4, 155.6)	0.54	63.39 (−167.83, 304.50)	0.57	0.56	62.48 (−161.42, 286.38)	0.58	−57.88 (−292.34, 176.58)	0.62	**0.07**
Model 2	−70.79 (−301.7, 160.1)	0.54	71.86 (−165.3, 309.1)	0.55	0.54	51.84 (−174.65, 278.33)	0.65	−65.30 (−301.11, 170.51)	0.58	**0.07**

DH, Duration of hospitalization; RDS, Respiratory distress syndrome; CRP, C–reactive protein. Model 1 was adjusted for age, sex, education level, BMI, and physical activity. Model 2 was adjusted age, sex, education level, BMI, physical activity, comorbidity, use of medication, and supplementation use. Data were obtained from the linear regression. Data are presented as β and 95% confidence interval. Significant associations with a P–value < 0.05 are bolded.

Furthermore, a positive association was observed between the ratio of N6/N3 ratio and D-dimer levels in tertile 3 compared to tertile 1 (β = 12.61; CI = 4.27, 20.96; *P-*value < 0.01) (*P*-trend < 0.01).

A marginally significant linear trend was observed over tertiles of (PUFA + MUFA)/SFA for DH (*P*-trend = 0.06) CRP, (*P*-trend = 0.06), and D-dimer (*P*-trend = 0.07).

### Associations between the quantity of fatty acids and signs and symptoms of COVID-19

Associations between the quantity of fatty acids and signs and symptoms of COVID-19 patients were presented in Table 3.

**Table 3:** Association of quantity of fatty acids with sign and symptoms of COVID-19 (*N* = 107).

**Variables**	**T2 (*n* = 35)**	***P-*value**	**T3 (*n* = 35)**	***P-*value**	***P*-trend**	**T2 (*n* = 35)**	***P-*value**	**T3 (*n* = 35)**	***P-*value**	***P*-trend**
**OR (95%CI)**										
	**PUFA**					**SFA**				
**Taste**										
Crude	1.14 (−0.58, 2.85)	0.19	1.24 (−0.47, 2.95)	0.15	0.15	1.22 (0.21, 6.81)	0.82	4.17 (0.74, 23.27)	0.10	0.10
Model 1	1.12 (−0.63, 2.89)	0.21	1.40 (−0.41, 3.22)	0.13	0.13	1.28 (0.22, 7.43)	0.78	4.09 (0.66, 25.56)	0.13	0.12
Model 2	1.25 (−0.45, 2.96)	0.15	1.54 (−0.24, 3.32)	0.15	0.09	1.21 (0.27, 8.42)	0.63	4.20 (0.27, 24.68)	0.11	0.10
**Smell**										
Crude	0.83 (−0.91, 2.58)	0.35	0.31 (−1.41, 2.03)	0.71	0.71	1.98 (0.34, 11.45)	0.44	1.40 (0.24, 8.13)	0.70	0.70
Model 1	1.12 (−0.61, 2.91)	0.22	0.61 (−1.27, 2.64)	0.51	0.50	1.58 (0.26, 9.53)	0.61	0.92 (0.14, 5.93)	0.93	0.92
Model 2	1.15 (−0.61, 2.92)	0.20	0.63 (−1.21, 2.48)	0.50	0.50	1.73 (0.29, 10.27)	0.54	0.95 (0.15, 5.95)	0.95	0.94
**Appetite**										
Crude	0.54 (−1.96, 1.88)	0.44	1.42 (−2.82, −0.01)	**0.04**	**0.04**	0.47 (0.11, 1.99)	0.31	0.63 (0.15, 2.65)	0.53	0.53
Model 1	0.34 (−1.78, 1.10)	0.64	1.42 (−2.91, 0.06)	**0.06**	**0.06**	0.47 (0.11, 2.03)	0.31	0.61 (0.13, 2.78)	0.52	0.53
Model 2	0.38 (−1.80, 1.04)	0.59	1.59 (−3.07, −0.10)	**0.03**	**0.03**	0.53 (0.12, 2.28)	0.39	0.65 (0.14, 2.91)	0.57	0.57
**Lethargy**										
Crude	1.45 (0.38, 5.48)	0.57	2.44 (0.66, 9.02)	0.18	0.18	1.67 (0.44, 6.26)	0.44	0.91 (0.4, 3.43)	0.89	0.89
Model 1	1.41 (0.36, 5.51)	0.61	2.46 (0.64, 9.33)	0.18	0.18	1.95 (0.50, 7.54)	0.33	1.06 (0.26, 4.30)	0.93	0.97
Model 2	1.82 (0.46, 7.09)	0.38	2.57 (0.69, 9.60)	0.15	0.15	1.99 (0.52, 7.58)	0.31	1.11 (0.28, 4.43)	0.87	0.89
**RDS**										
Crude	0.93 (0.32, 2.68)	0.90	0.33 (0.09, 1.19)	0.09	0.61	0.62 (0.20, 1.88)	0.40	0.51 (0.16, 1.62)	0.25	0.25
Model 1	1.07 (0.34, 3.35)	0.89	0.25 (0.05, 1.06)	0.06	0.07	0.60 (0.18, 1.95)	0.40	0.42 (0.12, 1.49)	0.18	0.17
Model 2	1.06 (0.33, 3.36)	0.91	0.24 (0.05, 1.06)	**0.06**	**0.07**	0.59 (0.17, 1.95)	0.38	0.40 (0.11, 1.46)	0.17	0.16
**Fever**										
Crude	0.69 (0.24, 1.97)	0.49	0.96 (0.33, 2.80)	0.94	0.80	1.13 (0.42, 3.08)	0.79	2.52 (0.82, 7.74)	0.10	0.11
Model 1	0.76 (0.25, 2.29)	0.63	1.15 (0.36, 3.68)	0.81	0.90	1.14 (0.40, 3.29)	0.79	2.82 (0.83, 9.61)	0.09	0.10
Model 2	0.67 (0.21, 2.07)	0.24	1.04 (0.30, 3.59)	0.94	0.95	1.15 (0.38, 3.44)	0.79	3.30 (0.89, 12.16)	**0.07**	**0.07**
Crude	0.71 (0.58, 1.85)	0.73	1.59 (0.58, 4.29)	0.36	0.37	0.78 (0.30, 2.05)	0.62	0.69 (0.26, 1.83)	0.46	0.46
Model 1	0.63 (0.23, 1.75)	0.38	1.58 (0.52, 4.79)	0.41	0.43	0.77 (0.28, 2.15)	0.62	0.62 (0.21, 1.84)	0.39	0.39
Model 2	0.60 (0.21, 1.69)	0.34	1.47 (0.47, 4.60)	0.50	0.52	0.78 (0.27, 2.18)	0.63	0.61 (0.20, 1.83)	0.38	0.38
**Contusion**										
Crude	1.15 (0.72, 1.84)	0.54	0.92 (0.33, 2.52)	0.87	0.91	1.26 (0.48, 3.26)	0.62	0.89 (0.34, 2.28)	0.81	0.81
Model 1	0.91 (0.34, 2.44)	0.85	1.39 (0.49, 3.93)	0.23	0.53	1.19 (0.44, 3.22)	0.72	0.78 (0.28, 2.20)	0.64	0.64
Model 2	0.91 (0.33, 2.51)	0.86	1.50 (0.51, 4.42)	0.45	0.45	1.09 (0.40, 2.99)	0.86	0.75 (0.26, 2.12)	0.58	0.58
**Confusion**										
Crude	0.70 (0.25, 1.94)	0.249	0.96 (0.34, 2.70)	0.94	0.98	0.77 (0.28, 2.08)	0.61	0.50 (0.17, 1.42)	0.19	0.19
Model 1	0.74 (0.25, 2.19)	0.59	1.23 (0.38, 3.94)	0.72	0.76	0.93 (0.32, 2.66)	0.89	0.65 (0.21, 2.04)	0.46	0.47
Model 2	0.72 (0.24, 2.17)	0.57	1.16 (0.35, 3.83)	0.79	0.72	0.91 (0.31, 2.65)	0.86	0.64 (0.20, 2.03)	0.45	0.46
**Sore throat**										
Crude	1.82 (0.67, 4.94)	0.24	1.04 (0.37, 2.92)	0.94	0.86	0.60 (0.22, 1.62)	0.31	0.60 (0.22, 1.62)	0.31	0.30
Model 1	2.68 (0.88, 8.12)	0.18	1.79 (0.55, 5.79)	0.32	0.60	0.62 (0.21, 1.80)	0.37	0.62 (0.20, 1.88)	0.40	0.40
Model 2	2.92 (0.92, 9.22)	0.16	2.21 (0.63, 7.64)	0.21	0.20	0.51 (0.16, 1.59)	0.25	0.57 (0.17, 1.82)	0.34	0.35
**Nausea**										
Crude	0.76 (0.29, 2.00)	0.58	1.48 (0.58, 3.78)	0.41	0.73	0.88 (0.34, 2.30)	0.80	1.12 (0.43, 2.88)	0.81	0.80
Model 1	0.82 (0.29, 2.29)	0.71	1.62 (0.56, 4.67)	0.37	0.37	0.75 (0.27, 2.08)	0.58	0.93 (0.32, 2.68)	0.91	0.91
Model 2	0.78 (0.27, 2.26)	0.65	1.56 (0.52, 4.67)	0.42	0.42	0.72 (0.25, 2.06)	0.54	0.93 (0.31, 2.74)	0.90	0.91
**Vomiting**										
Crude	0.76 (0.12, 4.58)	0.76	2.06 (0.41, 10.40)	0.38	0.31	0.56 (0.12, 2.56)	0.45	1.24 (0.34, 4.51)	0.74	0.72
Model 1	0.88 (0.15, 5.10)	0.89	2.52 (0.51, 12.46)	0.25	0.21	0.66 (0.13, 3.32)	0.61	1.60 (0.36, 7.12)	0.53	0.51
Model 2	0.49 (0.09, 2.50)	0.39	0.67 (0.15, 2.89)	0.59	0.55	0.66 (0.13, 3.35)	0.62	1.72 (0.39, 7.89)	0.47	0.47
**Headache**										
Crude	0.39 (0.15, 1.03)	**0.05**	0.94 (0.37, 2.39)	0.19	0.19	1.12 (0.43, 2.86)	0.81	1.25 (0.49, 3.21)	0.63	0.63
Model 1	0.37 (0.17, 1.37)	**0.05**	0.49 (0.17, 1.37)	0.17	0.17	0.87 (0.32, 2.39)	0.79	1.00 (0.35, 2.84)	0.99	0.99
Model 2	0.35 (0.12, 1.00)	**0.05**	0.47 (0.16, 1.39)	0.17	**0.07**	0.83 (0.30, 2.31)	0.72	0.98 (0.34, 2.82)	0.98	0.98
Crude	0.35 (0.13, 0.92)	**0.03**	0.42 (0.15, 1.10)	0.07	0.07	2.00 (0.77, 5.18)	0.15	1.41 (0.55, 3.62)	0.47	0.47
Model 1	0.29 (0.10, 0.8)	**0.02**	0.36 (0.12, 1.05)	0.06	0.06	1.78 (0.65, 4.83)	0.25	1.34 (0.47, 3.79)	0.57	0.58
Model 2	0.25 (0.08, 0.76)	**0.01**	0.30 (0.09, 0.93)	**0.03**	**0.03**	1.82 (0.65, 5.03)	0.24	1.37 (0.48, 3.89)	0.55	0.57
	**MUFA**					**Total fat**				
**Taste**										
Crude	7.38 (1.35, 40.16)	**0.02**	1.25 (0.23, 6.83)	0.79	0.79	3.66 (0.66, 20.18)	0.13	3.90 (0.69, 22.04)	0.12	0.12
Model 1	7.12 (1.30, 38.8)	**0.02**	1.23 (0.21, 7.11)	0.81	0.79	3.92 (071, 21.55)	0.11	4.10 (0.65, 25.79)	0.13	0.12
Model 2	5.76 (1.07, 30.84)	**0.04**	1.51 (0.26, 8.53)	0.63	0.59	3.48 (0.65, 18.65)	0.14	3.92 (0.95, 23.41)	0.13	0.12
**Smell**										
Crude	1.49 (0.26, 8.49)	0.65	0.40 (0.07, 2.28)	0.30	0.30	1.46 (0.25, 8.35)	0.67	1.13 (0.19, 6.67)	0.88	0.88
Model 1	1.56 (0.27, 8.88)	0.61	0.36 (0.06, 2.20)	0.27	0.28	1.35 (0.23, 7.70)	0.73	1.03 (0.15, 6.79)	0.97	0.88
Model 2	1.60 (0.28, 9.10)	0.59	0.34 (0.05, 2.05)	0.24	0.26	1.64 (0.28, 9.37)	0.57	1.02 (0.16, 6.52)	0.98	0.95
**Appetite**										
Crude	0.39 (0.09, 1.61)	0.19	0.35 (0.08, 1.48)	0.15	0.15	0.40 (0.10, 1.67)	0.21	0.31 (0.07, 1.30)	0.10	0.10
Model 1	0.42 (0.10, 1.75)	0.23	0.42 (0.09, 1.86)	0.25	0.25	0.41 (0.10, 1.71)	0.22	0.33 (0.07, 1.57)	0.16	0.15
Model 2	0.34 (0.08, 1.40)	0.13	0.44 (0.10, 1.90)	0.27	0.26	0.35 (0.08, 1.46)	0.15	0.31 (0.07, 1.42)	0.13	0.12
**Lethargy**										
Crude	0.56 (0.15, 2.11)	0.39	1.15 (0.30, 4.31)	0.83	0.83	0.39 (0.10, 1.43)	0.15	1.57 (0.42, 5.82)	0.49	0.51
Model 1	0.49 (0.13, 1.83)	0.29	1.16 (0.29, 4.54)	0.83	0.85	0.40 (0.11, 1.47)	0.17	1.74 (0.43, 7.06)	0.43	0.50
Model 2	0.49 (0.13, 1.86)	0.30	0.98 (0.25, 3.83)	0.98	0.95	0.41 (0.11, 1.51)	0.18	1.70 (0.43, 6.74)	0.44	0.51
**RDS**										
Crude	0.72 (0.23, 2.22)	0.57	0.72 (0.23, 2.22)	0.57	0.56	3.56 (1.28, 28.11)	0.06	3.58 (0.80, 18.60)	0.06	0.08
Model 1	0.71 (0.21, 2.55)	0.58	0.70 (0.20, 2.55)	0.60	0.56	4.73 (1.34, 28.70)	0.07	4.61 (0.90, 24.19)	0.07	0.09
Model 2	0.71 (0.21, 2.35)	0.58	0.71 (0.20, 2.50)	0.60	0.56	4.73 (1.26, 26.57)	**0.06**	4.68 (0.87, 23.78)	0.08	0.11
Crude	0.64 (0.22, 1.87)	0.42	0.74 (0.25, 2.17)	0.58	0.59	1.37 (0.48, 3.88)	0.54	1.27 (0.44, 3.61)	0.65	0.64
Model 1	0.69 (0.23, 2.05)	0.50	0.74 (0.23, 2.33)	0.61	0.84	1.45 (0.46, 3.87)	0.58	1.45 (0.46, 4.56)	0.52	0.50
Model 2	0.71 (0.22, 2.25)	0.52	0.63 (0.19, 2.07)	0.45	0.44	1.28 (0.42, 3.89)	0.65	1.50 (0.45, 4.95)	0.50	0.49
**Chills**										
Crude	1.26 (0.48, 3.30)	0.62	1.43 (0.54, 3.78)	0.46	0.46	1.32 (0.51, 3.40)	0.56	2.02 (0.74, 5.45)	0.16	0.16
Model 1	1.33 (0.48, 3.63)	0.57	1.23 (0.44, 3.46)	0.68	0.67	1.34 (0.51, 3.54)	0.54	1.96 (0.66, 5.76)	0.22	0.22
Model 2	1.26 (0.48, 3.30)	0.62	1.43 (0.54, 3.78)	0.46	0.01	1.32 (0.49, 3.56)	0.57	1.99 (0.66, 5.94)	0.21	0.21
**Contusion**										
Crude	0.63 (0.24, 1.62)	0.33	0.88 (0.34, 2.30)	0.80	0.81	1.18 (0.46, 3.00)	0.72	1.34 (0.52, 3.49)	0.53	0.53
Model 1	0.59 (0.22, 1.57)	0.29	0.77 (0.28, 2.13)	0.62	0.61	1.18 (0.45, 3.04)	0.73	1.26 (0.45, 3.53)	0.64	0.64
Model 2	0.64 (0.23, 1.76)	0.39	0.75 (0.27, 2.13)	0.60	0.59	1.19 (0.45, 3.18)	0.71	1.34 (0.47, 3.81)	0.58	0.58
**Confusion**										
Crude	1.30 (0.47, 3.59)	0.60	1.00 (0.35, 2.82)	1.00	1.00	1.78 (0.66, 4.79)	0.25	0.64 (0.21, 1.96)	0.44	0.48
Model 1	1.27 (0.43, 3.68)	0.65	0.98 (0.32, 2.99)	0.97	0.99	2.06 (0.71, 2.09)	0.17	0.60 (0.17, 2.09)	0.42	0.27
Model 2	1.36 (0.46, 4.03)	0.56	1.00 (0.32, 3.10)	1.00	0.99	2.34 (0.78, 7.01)	0.12	0.63 (0.17, 2.24)	0.47	0.33
**Sore throat**										
Crude	1.00 (0.37, 2.68)	1.00	0.76 (0.27, 2.11)	0.60	0.32	1.36 (0.52, 3.58)	0.52	0.49 (0.16, 1.47)	0.20	0.23
Model 1	0.82 (0.27, 2.47)	0.72	1.21 (0.40, 3.73)	0.72	0.61	1.58 (0.57, 4.35)	0.37	0.69 (0.21, 2.26)	0.69	0.63
Model 2	1.37 (0.46, 4.09)	0.56	1.09 (0.34, 3.46)	0.87	0.85	2.02 (0.68, 6.04)	0.20	0.71 (0.20, 2.44)	0.58	0.70
**Nausea**										
Crude	0.79 (0.30, 2.04)	0.62	0.89 (0.34, 2.28)	0.81	0.80	1.47 (0.53, 4.06)	0.45	0.98 (0.32, 2.98)	0.98	0.98
Model 1	0.83 (0.25, 2.25)	0.71	0.71 (0.26, 2.00)	0.52	0.84	1.60 (0.59, 4.32)	0.34	0.99 (0.33, 2.95)	0.99	0.95
Model 2	0.81 (0.29, 2.25)	0.68	0.69 (0.24, 1.98)	0.49	0.83	0.47 (0.16, 1.36)	0.16	0.80 (0.27, 2.32)	0.68	0.78
**Vomiting**										
Crude	1.77 (0.39, 8.09)	0.45	2.20 (0.50, 9.63)	0.29	0.29	0.34 (0.06, 1.89)	0.21	1.45 (0.41, 5.11)	0.56	0.50
Model 1	1.65 (0.32, 8.35)	0.54	1.76 (0.35, 8.82)	0.48	0.50	4.60 (0.80, 26.36)	0.08	2.38 (0.34, 16.32)	0.37	0.43
Model 2	1.33 (0.25, 7.15)	0.73	146 (0.28, 7.41)	0.67	0.65	3.98 (0.76, 20.71)	0.10	2.48 (0.51, 15.79)	0.23	0.27
Crude	0.44 (0.17, 1.15)	0.09	0.55 (0.21, 1.44)	0.23	0.23	0.75 (0.29, 1.91)	0.54	0.66 (0.25, 1.72)	0.40	0.40
Model 1	0.47 (0.17, 1.28)	0.14	0.47 (0.16, 1.34)	0.16	0.16	0.61 (0.22, 1.67)	0.34	0.55 (0.18, 1.63)	0.28	0.27
Model 2	0.46 (0.16, 1.30)	0.14	0.46 (0.15, 1.33)	0.15	0.14	0.56 (0.20, 1.56)	0.27	0.54 (0.18, 1.63)	0.28	0.27
**Chest pain**										
Crude	0.39 (0.15, 1.03)	0.05	0.62 (0.24, 1.62)	0.33	0.34	0.47 (0.18, 1.22)	0.12	0.75 (0.28, 1.94)	0.55	0.55
Model 1	0.37 (0.13, 1.02)	0.05	0.50 (0.17, 1.43)	0.20	0.19	0.38 (0.14, 1.05)	0.06	0.59 (0.20, 1.76)	0.35	0.31
Model 2	0.34 (0.12, 0.98)	0.04	0.48 (0.16, 1.41)	0.18	0.18	0.34 (0.12, 0.96)	**0.04**	0.57 (0.19, 1.74)	0.33	0.29

DH, Duration of hospitalization; RDS, Respiratory distress syndrome; CRP, C-reactive protein; PUFA, poly unsaturated fatty acid; MUFA, mono unsaturated fatty acid; SFA, saturated fatty acid; OR, odds ratio. Data were obtained from the binary regression. Model 1 was adjusted for age, sex, education level, BMI, and physical activity. Model 2 was adjusted age, sex, education level, BMI, physical activity, comorbidity, use of medication, and supplementation use. Data are presented as OR and 95% confidence interval. Significant associations with a P value < 0.05 are bolded.

After controlling for confounders, there were higher odds of appetite in the third tertile of PUFA (OR = 1.59, 95% CI = −3.07, −0.10; *P-*value = 0.03) (*P*-trend = 0.03).

Furthermore, marginally significant lower odds of RDS were shown in the third tertile of PUFA intake (OR = 0.24, 95% CI = 0.05, 1.06; *P-*value = 0.06) (*P*-trend = 0.07). Lower odds of headache were observed in second tertile of PUFA (OR = 0.35, 95% CI = 0.12, 1.00; *P-*value = 0.05) (*P*-trend = 0.07). Lower odds of chest pain were found in the second tertile (OR = 0.25, 95% CI = 0.08, 0.76; *P-*value = 0.01) and third tertile (OR = 0.30, 95% CI = 0.09, 0.93; *P-*value = 0.03) of PUFA intake (*P*-trend = 0.03). A marginally significant higher odds of fever were observed in the third tertile of SFA intake (OR = 3.30, 95% CI = 0.89, 12.16; *P-*value = 0.07) (*P*-trend = 0.07).

Marginally significant higher odds of RDS (OR = 4.73, 95% CI = 1.26, 26.57; *P-*value = 0.06), and lower odds of chest pain in tertial 2 (OR = 0.34, 95% CI = 0.12, 0.96; *P-*value = 0.04) were found in second tertile of total fat compared to tertial 1.

### Associations between quantity of fatty acids and sign and duration of hospitalization of COVID-19

Table 4 displays the associations between the quantity of fatty acids and the signs and duration of hospitalization in COVID-19 patients. A significant positive association between second tertile of PUFA intake and the duration of hospitalization compared to the first tertile (β = 1.95, 95% CI = 0.59, 3.32; *P-*value = 0.01). Furthermore, there was a direct association between SFA intake and duration of hospitalization (β = 1.86, 95% CI = 0.45, 3.28; *P-*value = 0.01). Additionally, a significant negative association was observed between MUFA intake and hospitalization duration in the second tertile (β = −196.07, 95% CI= −423.2, −31.05; *P-*value = 0.04). A positive association was observed between the intake of total fat and CRP levels in tertile 2 (β = 190.093, 95% CI = −373.9, −7.91; *P-*value = 0.04).

**Table 4:** Association of quantity of fatty acids with sign and duration of hospitalization of COVID-19 (*N* = 107).

**Variables**	**T2 (*n* = 35)**	***P-*value**	**T3 (*n* = 35)**	***P-*** **value**		***P*-trend**	**T2 (*n* = 35)**	***P-*value**	**T3 (*n* = 35)**	***P-*value**
β **(95%CI)**										
	**PUFA**					**SFA**				
**Recovery time (day)**										
Crude	0.88 (−1.44, 3.21)	0.45	0.40 (−1.93, 2.73)	0.73	0.73	1.62 (−0.67, 3.93)	0.82	−0.25 (−2.56, 2.04)	0.16	0.44
Model 1	0.92 (−1.23, 3.09)	0.40	−0.40 (−2.64, 1.83)	0.72	0.74	0.77 (−1.42, 2.96)	0.49	−1.06 (−3.33, 2.96)	0.35	0.31
Model 2	0.65 (−1.44, 2.76)	0.53	−0.55 (−2.72, 1.60)	0.61	0.64	1.14 (−0.96, 3.25)	0.28	−0.87 (−3.05, 1.29)	0.42	0.44
**DH (day)**										
Crude	2.00 (0.64, 3.36)	**0.04**	−8.88 (−1.36, 1.36)	1.00	1.00	1.69 (0.23, 3.02)	**0.02**	0.97 (−0.42, 2.36)	0.17	0.06
Model 1	2.08 (0.71, 3.45)	**0.03**	−0.09 (−1.50, 1.32)	0.89	0.94	1.68 (0.24, 3.12)	**0.02**	1.14 (−0.34, 2.63)	0.13	0.14
Model 2	1.95 (0.59, 3.32)	**0.01**	−0.15 (−1.56, 1.25)	0.83	0.92	1.86 (0.45, 3.28)	**0.01**	1.24 (−0.21, 2.70)	0.09	0.11
**CRP (mg/L)**										
Crude	41.60 (−153.45, 236.65)	0.67	−41.50 (−235.11, 152.10)	0.67	0.07	−161.3 (−354.4, 31.7)	0.10	−98.17 (−291.29, 94.94)	0.31	0.32
Model 1	29.42 (−164.09, 222.94)	0.76	−50.02 (−249.48, 149.43)	0.22	0.06	−138.16 (−325.51, 53.18)	0.15	−105.39 (−303.6, 92.85)	0.29	0.30
Model 2	23.19 (−167.04, 213.45)	0.81	−61.18 (−259.56, 137.16)	0.07	0.57	−142.45 (−331.14, 46.23)	0.13	−105.27 (−299.8, 89.2)	0.28	0.30
**D-dimer (ng/ml)**										
Crude	3.49 (−226.81, 233.7)	0.97	14.12 (−214.48, 242.72)	0.90	0.97	141.12 (−86.5, 368.7)	0.22	168.1 (−59.50, 395.7)	0.14	0.14
Model 1	−14.93 (−243.06, 213.1)	0.89	−29.76 (−264.8, 205.3)	0.80	0.80	64.82 (−161.9, 291.6)	0.57	57.45 (−177.5, 22.43)	0.63	0.63
Model 2	−19.56 (−247.51, 208.37)	0.86	−44.54 (−28.23, 193.13)	0.71	0.71	76.40 (−150.9, 303.7)	0.59	64.01 (−170.3, 298.3)	0.60	0.51
	**MUFA**					**Total fat**				
**Recovery time (day)**										
Crude	0.90 (−1.41, 3.21)	0.44	−0.86 (−3.19, 1.45)	0.46	0.46	1.29 (−1.01, 3.59)	0.27	1.01 (−1.32, 3.35)	0.60	0.61
Model 1	1.15 (−1.04, 3.35)	0.30	0.15 (−2.15, 2.46)	0.89	0.90	0.67 (−1.48, 2.82)	0.54	−0.15 (−2.48, 2.16)	0.89	0.92
Model 2	1.13 (−1.04, 3.31)	0.30	0.27 (−1.96, 2.51)	0.84	0.80	0.80 (−1.29, 2.89)	0.45	−0.27 (−2.49, 1.95)	0.81	0.85
**DH (day)**										
Crude	−0.01 (−1.44, 1.41)	0.98	−0.15 (−1.58, 1.29)	0.83	0.83	0.78 (−0.62, 2.19)	0.27	0.26 (−1.17, 1.69)	0.72	0.71
Model 1	−211.34 (−430.10, 7.42)	**0.05**	−166.09 (−390.31, 60.12)	0.15	0.14	0.73 (−0.69, 2.16)	0.31	0.22 (−1.31, 1.76)	0.77	0.73
Model 2	−196.07 (−423.2, 31.05)	**0.04**	−85.22 (−312.36, 141.90)	0.13	0.46	0.64 (−0.78, 2.07)	0.37	0.16 (−1.35, 1.67)	0.83	0.80
Crude	−83.400 (−278.31, 111.51)	0.54	−60.85 (−255.76, 134.05	0.40	0.21	−229.01 (−417.93, −40.06)	**0.01**	−165.52 (−357.18, 26.12)	0.09	0.09
Model 1	−112.37 (−300.6, 75.91)	0.24	−47.06 (−241.7, 147.64)	0.63	0.22	−216.79 (−400.0, −33.57)	**0.02**	−160.20 (−357.77, 37.36)	0.11	0.06
Model 2	−97.20 (−284.20, 89.7)	0.30	–−66.83 (−259.6, 125.9)	0.49	0.48	190.93 (−373.9, −7.91)	**0.04**	−159.18 (−353.8, 35.51)	0.10	0.30
**D-dimer (ng/ml)**										
Crude	−113.93 (−343.1, 115.33)	0.33	−44.11 (−274.96, 186.74)	0.70	0.71	14.73 (−213.72, 243.19)	0.89	45.10 (−186.64, 276.85)	0.70	0.90
Model 1	−68.90 (−29.4, 155.6)	0.54	63.39 (−167.83, 304.50)	0.57	0.56	−32.24 (−225.8, 188.40)	0.77	−77.98 (−315.9, 159.94)	0.52	0.52
Model 2	−70.79 (−301.7, 160.1)	0.54	71.86 (−165.3, 309.1)	0.55	0.54	76.40 (−150.9, 303.7)	0.49	64.01 (−170.3, 298.3)	0.81	0.50

DH, Duration of hospitalization; RDS, Respiratory distress syndrome; CRP, C-reactive protein; PUFA, poly unsaturated fatty acid; MUFA, mono unsaturated fatty acid; SFA, saturated fatty acid. Model 1 was adjusted for age, sex, education level, BMI, and physical activity. Model 2 was adjusted age, sex, education level, BMI, physical activity, comorbidity, use of medication, and supplementation use. Data were obtained from the linear regression. Data are presented as β and 95% confidence interval. Significant associations with a P-value < 0.05 are bolded.

## Discussion

To the best of our knowledge, this study is the first that investigated associations between the quantity and quality of dietary fats and symptoms and hospitalization duration among COVID-19 patients. The main findings were that a higher N6/N3 ratio was linked to greater odds of respiratory RDS and elevated D-dimer levels, while being correlated with lower odds of fever. While RDS odds increased over The VitE/PUFA ratio, chill odds declined. In contrast, the (PUFA + MUFA)/SFA ratio was associated with reduced odds of chest pain, DH time, CRP, and D-dimer levels. Furthermore, higher PUFA intake was linked to lower odds of poor appetite, RDS, and headaches, whereas increased SFA intake was associated with higher odds of fever. Additionally, elevated CSI levels were linked to prolonged DH time.

The quality and quantity of dietary fats play a crucial role in regulating inflammation levels in the body. A recent study examined the impact of dietary fats on immune responses related to COVID-19 and found that SFAs were linked to pro-inflammatory effects, potentially exacerbating complications such as cytokine storms and acute respiratory distress syndrome (ARDS). In contrast, PUFAs may help modulate immune responses and reduce inflammation. Notably, omega-3 PUFAs, including docosapentaenoic acid (DPA), eicosapentaenoic acid (EPA), and docosahexaenoic acid (DHA), exhibit anti-inflammatory properties. These fatty acids may alleviate COVID-19 symptoms by reducing pro-inflammatory cytokines and promoting a balanced immune response (22). This immune modulation could reduce the risk of severe conditions such as ARDS and cytokine storms associated with COVID-19. Furthermore, an increased intake of N3 PUFAs can lower the N6/N3 ratio, enhance anti-inflammatory processes, and mitigate the severity of COVID-19 symptoms (22). Our findings align with the previous research, demonstrating that higher SFAs intake is associated with an increased fever. Additionally, a positive linear trend was observed between CSI and DH, highlighting their potentially harmful pro-inflammatory effects on systemic inflammation. Also, an elevated N6/N3 ratio was linked to more severe complications, including RDS and elevated D-dimer levels while a direct correlation with fever was found. This suggests that the N6/N3 ratio may influence more critical inflammatory and thrombotic pathways. Additionally, increasing PUFAs intake, particularly omega-3 PUFAs, may help reduce the risk of severe respiratory complications, as a decreasing trend was observed between PUFA intake and RDS.

It is important to acknowledge that research on dietary intake and its correlation with COVID-19 symptoms remains limited. While strong evidence supports the beneficial effects of omega-3 PUFAs (15), there is a notable gap in research regarding the role of omega-6 fatty acids. ARDS is characterized by excessive immune cell activation and systemic inflammation, suggesting that anti-inflammatory diets may help alleviate symptoms (16). Clinical studies have shown that omega-3 PUFAs can play a beneficial role on managing ARDS (16). Additionally, omega-3 PUFAs supplementation was associated with reduced serum CRP levels in several medical conditions, including cardiovascular diseases, inflammatory disorders, and autoimmune diseases (17, 18). A systematic review found that omega-3 PUFAs supplementation significantly lowered CRP serum levels in COVID-19 patients compared to the control group (19). In addition, a recent study demonstrated a positive correlation between the dietary inflammatory index and elevated CRP and erythrocyte sedimentation rate (ESR) in COVID-19 patients (20). A 2024 cross-sectional study examined the relationship between dietary omega-3 PUFAs consumption and COVID-19 symptoms in 250 patients found an inverse correlation (23). Similarly, a recent 2024 study involving 43,155 American adults reported that higher intake of dietary omega-3 and omega-6 PUFAs was associated with lower levels of various inflammatory biomarkers (24). Furthermore, an Iranian study found that patients receiving omega-3 PUFAs supplementation showed significant clinical improvement in nearly all symptoms except for bodily pain, fatigue, appetite loss, and olfactory symptoms. Notably, omega-3 PUFAs supplementation significantly reduced CRP levels compared to the control group after therapy (25). Our findings are consistent with previous research, confirming a decreasing linear trend between PUFA intake and poor appetite, RDS, and headaches. Additionally, the analysis revealed a decreasing linear trend between the (PUFA+MUFA)/SFA ratio and conditions including chest pain, DH, CRP, and D-dimer, highlighting the beneficial effects of unsaturated fatty acids compared to saturated fatty acids.

On the other hand, a study reported no significant correlation between the N6/N3 ratio and inflammatory biomarkers (24). Furthermore, two studies found that omega-3 PUFA supplementation did not lead to a reduction in CRP levels among healthy individuals and HIV-infected adults (26, 27). This inconsistency may be attributed to low-dose omega-3 PUFA supplementation and various disease backgrounds of the study populations (26, 27).

A study on 600 Iranian COVID-19 patients found that adherence to the Mediterranean diet was associated with a lower incidence of symptoms including fever, cough, diarrhea, taste changes, and a reduced pneumonia severity index. Additionally, patients following the Mediterranean diet showed lower CRP levels. Given its anti-inflammatory properties, the Mediterranean diet may help alleviate symptoms and reduce serum inflammatory markers in COVID-19 patients, highlighting the need for further clinical trials to confirm these findings (21). Another study indicated that a healthy dietary pattern including eggs, liquid oils, vegetables, fruits, and nuts, exhibits significant anti-inflammatory effects. This dietary approach has been associated with a decreased risk of severe health outcomes, shorter durations of hospitalization, faster recovery rates, and reduced symptoms such as dyspnea and fatigue. These findings suggest that a diet rich in healthier fats and whole foods may mitigate inflammation and enhance resilience against COVID-19 (28). The alignment between these studies and our findings highlights associations between dietary fats and COVID-19 symptom severity. The Mediterranean diet study supports our result that higher quality fats PUFA, (PUFA + MUFA)/SFA and VitE/PUFA were linked to fewer symptoms, including chills, chest pain, poor appetite, and headache. Furthermore, the study on healthy dietary patterns reinforces our findings that higher PUFA and MUFA intake were linked to improved outcomes, suggesting that healthier fats may play a crucial role in reducing symptom severity and hospitalization duration.

Various mechanisms may be involved in the role of dietary fat intake in COVID-19 symptoms. SFAs contribute to the onset of various diseases, particularly cardiovascular diseases and inflammation (14, 29). The existing evidence shows that the increased levels of inflammatory cytokines may play a role in the pain occurrence (30). SFAs have been increasingly linked to the activation of inflammatory pathways including toll-like receptors, protein kinase C, reactive oxygen species, NOD-like receptors, and endoplasmic reticulum stress (29). MUFAs have been extensively studied for their protective effects, notably in cardiovascular and inflammation-related diseases (31, 32). However, elevated MUFA levels may not always be beneficial for inflammation. In patients with chronic kidney disease, an elevated MUFA/SFA ratio in blood lipids, linked to increased Stearoyl-CoA Desaturase-1 activity, has been correlated with high levels of circulating CRP, suggesting an exacerbation of inflammation (33). *In vitro* and *in vivo* research suggests that the uptake of saturated and unsaturated fatty acids influences the uptake of foreign pathogens by macrophages (34). The phagocytic activity of immune cells is positively correlated with total PUFA content and omega-3 PUFAs, while it is negatively correlated with palmitic acid content and the SFA/PUFA ratio (34). Administering a mixture of omega-3 PUFAs (DHA + EPA) at a dose of 1.5 g/day has improved phagocytic activity in neutrophils and monocytes (34). Furthermore, omega-3 PUFAs exhibit anti-inflammatory properties through various mechanisms, including the suppression of arachidonic acid-derived eicosanoid mediators, which are known for their pro-inflammatory effects (35).

The enzymatic oxidation of EPA and DHA contributes to inflammation improvement (36). The evidence shows that omega-3 and omega-6 PUFAs engage in competitive interactions at the cyclooxygenase and lipoxygenase sites, producing varied eicosanoids, including prostaglandins and leukotrienes (37). Conversely, the metabolism of EPA and DHA within N3 PUFAs produces prostaglandin E3 and leukotriene B5, demonstrating anti-inflammatory and antiplatelet aggregation properties (38). Regarding pain modulation, DHA enhance glutaminergic and serotonergic synaptic activity, inhibit histone deacetylation and mitigate pain progression (39, 40). Furthermore, N3 may exert analgesic effect by reducing D- and E-series resolving and arachidonic acid derivatives which are known to suppress pain receptor activity, including transient receptor potential vanilloid 1 (41, 42).

This study has several strengths. Notably, to the best of our knowledge, it is the first investigation into the relationship between dietary fat quality and quantity and the symptoms and hospitalization duration in individuals with COVID-19. Furthermore, the comprehensive analysis of both the quality and quantity of dietary fats provides valuable insights into dietary recommendations for managing COVID-19. However, the limitations need to be considered when interpreting these findings. Firstly, due to the cross-sectional study design, causality cannot be established. Secondly, the use of self-reported dietary questionnaires may have introduced reporting bias, potentially affecting the accuracy of dietary intake assessments. In addition to controlling for confounders in this study, there may have been residual confounding. As a result, further research, including longitudinal and interventional studies, are necessary to elucidate the relationship between dietary fat intake and COVID-19 severity and hospitalization duration.

## Conclusion

Our findings suggest that the quality of specific fatty acids is associated with various health outcomes, particularly in the context of COVID-19. A higher N6/N3 ratio was associated with increased odds of RDS while correlated with lower odds of fever. Additionally, higher D-dimer levels linked to a higher N6/N3 ratio. While the RDS risk increased over the VitE/PUFA ratio tertiles, chills decreased. Conversely, the chest pain, DH time, CRP, and D-dimer reduced over the (PUFA + MUFA)/SFA ratio tertiles. Furthermore, higher PUFA intake was associated with decreasing odds of poor appetite, RDS, and headaches, while increased SFA intake correlated with higher odds of fever. Additionally, higher CSI levels were associated with increased DH time. However, the evidence regarding the associations between different fatty acids and COVID-19 is very limited. As a result, further longitudinal studies are required to expand our understanding of the role of various fatty acids in COVID-19 progression and recovery.

## Data Availability

The raw data supporting the conclusions of this article will be made available by the authors, without undue reservation.
