# Supplementary material for: The association between dietary fat quality and quantity and hospitalization duration in COVID-19 in Iranian patients: a cross-sectional study
Source: Front Nutr. 2025 Apr 22;12:1551760. doi: 10.3389/fnut.2025.1551760 (PMC12052533; doi:10.3389/fnut.2025.1551760)
Supplement: Supplementary file 1 [file Table_1.docx]

| Supplementary Table 1. Characteristics of the study participants (N=107 ). | | |
| --- | --- | --- |
| Continuous variables | **Mean** | **SD** |
| Energy (kcal/d) | 2003.79 | 14.9 |
| Age (year) | 46.21 | 1.10 |
| Weight (kg) | 86.75 | 1.31 |
| Height (cm) | 170.92 | 0.78 |
| BMI (kg/m^2^) | 29.36 | 0.35 |
| DH (day) | 4.18 | 0.29 |
| Categorical variable | **Number** | **%** |
| Sex |  |  |
| Female | 36 | 33.6 |
| Male | 71 | 66.3 |
| Marriage status | | |
| Single | 11 | 10.5 |
| Education level | | |
| University educated | 55 | 53.4 |
| Job group | | |
| Office work | 23 | 21.9 |
| Employe | 4 | 3.8 |
| Educational work | 8 | 7.6 |
| Unemployed | 10 | 9.5 |
| Self employment | 62 | 57.9 |
| PA |  |  |
| Low | 98 | 93.3 |
| Moderate | 4 | 3.8 |
| Severe | 3 | 2.9 |
| Comorbidity |  |  |
| Cardiovascular | 10 | 9.5 |
| Renal | 5 | 4.8 |
| Diabetes | 6 | 5.7 |
| Thyroidysm | 7 | 6.7 |
| Supplements intake |  |  |
| Yes | 30 | 28.6 |
| Antibiotic use |  |  |
| Yes | 41 | 39 |
| Abbreviation: SD, Standard deviation; BMI, Body Mass Index; DH, Duration of hospitalization; PA, Phyisical activity.  Values are mean (± SD) for continuous variables and number (%) for categorical variables. | | |

| Supplementary Table 2. The association of signs, symptoms and duration of hospitalization in tertiles of dietary fat quality indices (N=107). | | | | | | | | | | |
| --- | --- | --- | --- | --- | --- | --- | --- | --- | --- | --- |
| Variables | **(PUFA +MUFA)/ SFA** | | | | | **VitE/PUFA** | | | | |
|  | **T_1_** | **T_2_** | **T_3_** | **P-value** | **P-value*** | **T_1_** | **T_2_** | **T_3_** | **P-value** | **P-value*** |
|  | <2.6 | 2.6-3.4 | >3.4 |  |  | **<0.89** | **0.89-1.03** | **>1.03** |  |  |
| Hospitalization days |  | | | | | | | | | |
| DH (day) | 4.20±2.86 | 4.71±3.56 | 3.62±2.67 | 0.33 | 0.60 | 4.34±3.73 | 4.11±2.84 | 5.40±4.25 | 0.93 | 0.61 |
| Recovery time (day) | 5.48±4.03 | 7.17±6.26 | 6.20±4.45 | 0.37 | 0.69 | 6.65±5.35 | 6.8±5.37 | 5.40±5.01 | 0.44 | 0.20 |
| Signs |  | | | | | | | | | |
| CRP (mg/L) | 74.0±115.3 | 74.4±126.1 | 43.8±34.3 | 0.42 | **0.03** | 57.2±128.5 | 78.7±110.3 | 51.2±40.4 | 0.55 | 0.40 |
| D-dimer  (ng/ml) | 9.28±13.86 | 7.43±10.39 | 9.28±12.95 | 0.89 | 0.13 | 4.97±9.92 | 10.9±12.8 | 9.24±12.42 | 0.38 | 0.63 |
| Symptoms |  | | | | | | | | | |
| Smell (poor) | 18(51.4) | 14(40.0) | 22(62.9) | 0.33 | 0.67 | 17(48.6) | 17(48.6) | 20(57.1) | 0.44 | 0.61 |
| Taste (poor) | 10(28.6) | 11(31.4) | 12(34.3) | 0.50 | 0.49 | 10(28.6) | 9(25.7) | 14(40.0) | 0.59 | 0.40 |
| Appetite (poor) | 13(37.1) | 13(37.1) | 13(37.1) | 1.00 | 0.48 | 17(48.6) | 9(25.7) | 13(37.1) | 0.13 | 0.39 |
| Lethargy (yes) | 11(31.4) | 14(40.0) | 14(40.0) | 0.51 | 0.09 | 7(20.0) | 5(14.3) | 6(17.1) | 0.33 | 0.51 |
| Chest pain (yes) | 24(86.6) | 14(40.0) | 16(45.7) | **0.04** | 0.72 | 17(48.6) | 20(57.1) | 17(48.6) | 0.71 | 0.35 |
| Headache (yes) | 23(65.7) | 15(42.9) | 16(45.7) | 0.11 | 0.53 | 17(48.6) | 19(54.3) | 18(54.1) | 0.89 | 0.56 |
| Vomiting (yes) | 5(14.3) | 2(5.7) | 7(20.0) | 0.20 | 0.80 | 8(22.9) | 4(11.4) | 2(5.7) | 0.09 | 0.15 |
| Nausea (yes) | 17(48.6) | 12(34.3) | 16(45.7) | 0.44 | 0.74 | 19(54.3) | 11(31.4) | 15(42.9) | 0.15 | 0.85 |
| Sore throat (yes) | 9(25.7) | 14(40.0) | 11(31.4) | 0.43 | 0.18 | 10(28.6) | 10(28.6) | 14(40.0) | 0.49 | 0.83 |
| Confusion (yes) | 8(22.9) | 9(25.7) | 15(42.9) | 0.14 | **0.02** | 10(28.6) | 13(37.1) | 9(25.7) | 0.55 | 0.39 |
| Fever (yes) | 30(85.7) | 23(65.7) | 23(65.7) | 0.09 | 0.87 | 31(88.6) | 19(54.3) | 26(74.3) | **0.006** | 0.49 |
| Chills (yes) | 23(65.7) | 17(48.6) | 25(71.4) | 0.12 | 0.64 | 29(82.9) | 17(48.6) | 19(54.3) | **0.007** | **0.03** |
| RDS (yes) | 7(20.0) | 6(17.1) | 10(28.6) | 0.48 | 0.13 | 3(8.6) | 11(31.4) | 9(25.7) | **0.05** | 0.69 |
| Variables | **CSI** | | | | | **N6/N3** | | | | |
|  | T_1_ | T_2_ | T_3_ | P-value | P-value* | T_1_ | T_2_ | T_3_ | P-value | P-value* |
|  | <26.58 | 26.58-32.13 | >32.13 |  |  | <25.26 | 25.26-38.23 | >38.23 |  |  |
| Hospitalization days |  | | | | | | | | | |
| DH (day) | 3.50±2.32 | 4.30±354 | 4.71±3.13 | 0.24 | 0.23 | 4.37±3.74 | 4.51±2.80 | 3.65±2.54 | 0.46 | 0.17 |
| Recovery time (day) | 6.14±5.08 | 6.69±5.36 | 6.0±4.68 | 0.83 | 0.23 | 7.11±5.50 | 5.97±5.10 | 5.77±4.40 | 0.48 | 0.60 |
| Signs |  | | | | | | | | | |
| CRP (mg/L) | 91.9±166.6 | 50.2±29.60 | 50.2±29.6 | 0.23 | 0.94 | 97.4±149.1 | 42.2±41.80 | 43.8±32.17 | **0.05** | **0.07** |
| D-dimer (ng/ml) | 9.19±12.28 | 9.70±11.11 | 7.31±12.9 | 0.85 | 0.08 | 6.86±9.95 | 8.04±11.7 | 12.2±14.7 | 0.48 | **0.03** |
| Symptoms |  | | | | | | | | | |
| Smell (poor) | 18(52.9) | 16(44.4) | 20(57.1) | 0.78 | 0.11 | 17(48.6) | 19(54.3) | 18(51.4) | 0.98 | 0.39 |
| Taste (poor) | 12(35.3) | 12(35.3) | 9(25.7) | 0.71 | 0.52 | 13(37.1) | 8(22.9) | 12(34.3) | 0.39 | 0.65 |
| Appetite (poor) | 14(41.2) | 13(36.1) | 12(34.3) | 0.85 | 0.16 | 14(40.0) | 15(42.9) | 10(28.6) | 0.14 | 0.22 |
| Lethargy (yes) | 7(20.6) | 4(11.1) | 7(20.0) | 0.82 | 0.60 | 7(20.2) | 6(17.1) | 5(14.3) | 0.62 | 0.31 |
| Chest pain (yes) | 20(58.8) | 15(41.7) | 19(54.3) | 0.32 | 0.60 | 20(57.1) | 17(48.6) | 17(48.6) | 0.71 | 0.66 |
| Headache (yes) | 20(58.8) | 14(38.9) | 20(57.1) | 0.17 | 0.33 | 18(51.4) | 18(51.4) | 18(51.4) | 1.00 | 0.62 |
| Vomiting (yes) | 3(8.8) | 4(11.1) | 7(20.0) | 0.35 | **0.07** | 6(17.1) | 3(8.6) | 5(14.3) | 0.56 | 0.99 |
| Nausea (yes) | 13(38.2) | 15(41.7) | 17(48.6) | 0.67 | 0.71 | 16(45.7) | 14(40.0) | 15(42.9) | 0.89 | 0.60 |
| Sore throat (yes) | 11(32.4) | 13(36.1) | 10(28.6) | 0.79 | 0.12 | 11(31.4) | 11(31.4) | 12(34.3) | 0.95 | 0.48 |
| Confusion (yes) | 10(29.4) | 11(30.6) | 11(31.4) | 0.98 | 0.97 | 12(34.3) | 7(20.0) | 13(37.1) | 0.24 | 0.44 |
| Fever (yes) | 24(70.6) | 25(69.4) | 27(77.1) | 0.73 | 0.35 | 30(85.7) | 25(71.4) | 21(60.0) | **0.05** | 0.75 |
| Chills (yes) | 22(67.4) | 22(61.1) | 21(6.0) | 0.91 | 0.28 | 23(65.7) | 22(62.9) | 20(57.1) | 0.75 | 0.17 |
| RDS (yes) | 7(20.6) | 8(22.2) | 8(22.9) | 0.97 | 0.92 | 5(14.3) | 6(17.1) | 12(34.3) | 0.09 | 0.78 |
| Abbreviation: SD, Standard deviation; DH, Duration of hospitalization; RDS, Respiratory distress syndrome; CRP, C-reactive protein; Quantitative variables as means ± SD obtained from the independent t-test.  Categorical variables N (%) obtained from the Chi-square analysis. Significant associations with a *P-value* < 0.07 are bolded. *P-value** adjusted for age, BMI, energy, physical activity and education level. | | | | | | | | | | |

| Supplementary Table 3. The association of signs, symptoms and duration of hospitalization in tertiles of dietary fat quantity (N=107). | | | | | | | | | | | | | | | | |
| --- | --- | --- | --- | --- | --- | --- | --- | --- | --- | --- | --- | --- | --- | --- | --- | --- |
| Variables |  | | | |  | | | |  | | | |  | | | |
|  | **linolenic acid** | | ***P-value*** | ***P-value**** | **Oleic acid** | | **p-value** | **P-value***** | **MUFA** | | ***P-value*** | ***P-value**** | **Cholesterol** | | ***P-value*** | ***P-value**** |
|  | **<0.73** | **>0.73** |  |  | **<33.68** | **≥33.68** |  |  | **<35.89** | **≥35.89** |  |  | **<183.60** | **≥183.60** |  |  |
| Hospitalization days |  | | | | | | | | | | | | | | | |
| DH (day) | 3.46±2.27 | 4.88±3.56 | **0.01** | **0.01** | 4.15±3.12 | 4.20±3.03 | 0.90 | 0.87 | 4.19±3.17 | 4.16±2.98 | 0.51 | 0.42 | 3.86±2.63 | 4.49±3.43 | 0.29 | 0.92 |
| Recovery time (day) | 5.42±1.05 | 7.13±5.71 | 0.08 | 0.29 | 6.34±5.14 | 6.22±4.92 | 0.92 | **0.07** | 5.96±4.82 | 6.60±5.21 | 0.97 | 0.29 | 6.25±4.97 | 6.32±5.10 | 0.94 | 0.22 |
| Signs |  | | | | | | | | | | | | | | | |
| CRP(mg/L) | 60.81±101.54 | 65.56±96.1 | 0.83 | 0.19 | 76.62±137.10 | 51.2±36.24 | 0.25 | 0.97 | 79.91±140.17 | 49.7±36.55 | 0.17 | 0.74 | 81.84±132.90 | 45.70±39.87 | 0.10 | 0.42 |
| D-dimer  (ng/ml) | 8.45±13.15 | 8.89±11.35 | 0.90 | 0.21 | 9.95±11.97 | 7.37±12.05 | 0.47 | **0.05** | 10.35±12.07 | 7.09±11.85 | 0.36 | 0.25 | 6.70±10.29 | 11.1±13.52 | 0.21 | **0.04** |
| Symptoms |  | | | | | | | | | | | | | | | |
| Smell (poor) | 25(48.1) | 27(50.9) | 0.38 | 0.65 | 24(46.1) | 28(52.8) | 0.24 | 0.58 | 25(48.1) | 27(50.9) | 0.38 | 0.57 | 29(55.8) | 23(43.4) | 0.19 | 0.11 |
| Taste (poor) | 19(36.5) | 32(60.4) | 0.14 | 0.92 | 18(34.6) | 30(56.6) | 0.38 | 0.87 | 18(34.6) | 29(54.7) | 0.54 | 0.89 | 19(36.5) | 31(58.8) | 0.24 | 0.25 |
| Appetite (poor) | 15(28.8) | 25(47.2) | 0.09 | **0.06** | 18(34.4) | 23(43.4) | 0.30 | 0.28 | 19(36.5) | 22(41.5) | 0.45 | 0.28 | 20(38.5) | 20(37.7) | 0.39 | 0.41 |
| Lethargy (yes) | 23(44.2) | 16(30.5) | **0.07** | **0.07** | 18(34.5) | 21(39.6) | 0.67 | 0.72 | 19(36.5) | 20(37.7) | 0.85 | 0.64 | 17(32.7) | 22(56.4) | 0.61 | 0.37 |
| Chest pain (yes) | 26(50.0) | 28(52.8) | 0.46 | 0.78 | 30(57.7) | 24(45.3) | 0.14 | 0.42 | 32(61.5) | 22(41.5) | **0.03** | 0.08 | 28(53.8) | 26(49.1) | 0.38 | 0.96 |
| Headache (yes) | 24(46.2) | 30(56.6) | 0.19 | 0.30 | 28(53.8) | 26(49.1) | 0.38 | 0.10 | 30(57.7) | 24(45.3) | 0.14 | 0.10 | 28(53.8) | 26(49.1) | 0.38 | 0.68 |
| Vomiting (yes) | 3(5.8) | 11(20.8) | **0.02** | 0.19 | 6(11.5) | 8(15.1) | 0.40 | 0.76 | 6(11.5) | 8(15.1) | 0.40 | 0.34 | 6(11.5) | 8(15.1) | 0.40 | 0.08 |
| Nausea (yes) | 18(34.6) | 27(50.9) | **0.06** | 0.16 | 22(42.3) | 23(43.4) | 0.53 | 0.79 | 22(42.3) | 23(43.4) | 0.53 | 0.40 | 21(40.4) | 24(45.3) | 0.37 | 0.60 |
| Sore throat (yes) | 18(34.6) | 16(30.2) | 0.39 | 0.66 | 17(32.7) | 17(32.1) | 0.55 | 0.97 | 17(32.1) | 17(32.1) | 0.55 | 0.38 | 17(32.7) | 17(32.1) | 0.55 | 0.40 |
| Confusion (yes) | 17(32.7) | 15(28.3) | 0.39 | 0.59 | 16(30.8) | 16(30.2) | 0.55 | 0.42 | 17(32.8) | 15(28.3) | 0.39 | 0.53 | 17(32.7) | 15(28.3) | 0.39 | 0.90 |
| Fever (yes) | 32(61.5) | 44(83.0) | **0.01** | 0.08 | 38(73.1) | 38(71.7) | 0.52 | 0.78 | 39(75) | 37(69.8) | 0.35 | 0.35 | 38(73.1) | 38(71.7) | 0.62 | 0.47 |
| Chills (yes) | 29(55.8) | 36(67.9) | 0.14 | 0.11 | 30(57.7) | 35(66) | 0.24 | **0.06** | 32(61.5) | 33(62.3) | 0.54 | **0.04** | 33(63.5) | 32(60.4) | 0.45 | 0.45 |
| RDS (yes) | 17(32.7) | 6(11.3) | **0.007** | 0.09 | 13(25) | 10(18.9) | 0.30 | 0.83 | 13(25) | 10(18.9) | 0.30 | 0.85 | 12(23.1) | 11(20.8) | 0.47 | 0.64 |
|  | **Linoleic acid** | | ***p-value*** | ***P*-value*** | **PUFA** | | ***p-value*** | ***P*-value*** | **SFA** | | **p-value** | **P*-value** | **Total fat** | | ***p-value*** | ***P*-value*** |
|  | **<23.03** | **≥23.03** |  |  | **<25.80** | **≥25.80g/d** |  |  | **<19.27g/d** | **≥19.27g /d** |  |  | **<87.27g/d** | **≥87.27g/d** |  |  |
| Variables |  | | | | | | | | | | | | | | | |
| Hospitalization days |  | | | | | | | | | | | | | | | |
| DH (day) | 4.11±3.19 | 4.24±2.96 | 0.45 | 0.72 | 3.98±2.75 | 4.37±5.35 | 0.61 | 0.62 | 4.01±3.07 | 4.33±3.08 | 0.59 | 0.49 | 4.00±2.59 | 4.35±3.48 | 0.55 | 0.56 |
| Recovery time (day) | 6.65±5.50 | 5.92±4.50 | 0.82 | 0.31 | 6.03±4.71 | 6.52±5.32 | 0.51 | 0.45 | 6.28±5.26 | 6.28±4.80 | 0.99 | 0.08 | 5.67±4.20 | 6.88±5.67 | 0.21 | 0.29 |
| Sign |  | | | | | | | | | | | | | | | |
| CRP (mg/L) | 62.79±98.72 | 63.82±98.85 | 0.96 | 0.39 | 66.36±100.24 | 60.40±97.30 | 0.79 | 0.40 | 78.18±135.94 | 49.87±39.0 | 0.20 | 0.44 | 84.16±136.92 | 45.42±37.09 | 0.08 | 0.34 |
| D-dimer  (ng/ml) | 7.75±10.85 | 9.53±12.96 | 062 | 0.20 | 8.73±11.21 | 8.71±12.97 | 0.99 | 0.27 | 10.80±12.42 | 6.24±11.1 | 0.20 | 0.37 | 11.67±12.49 | 5.20±10.50 | **0.06** | 0.87 |
| Symptoms |  | | | | | | | | | | | | | | | |
| Smell (poor) | 25(48.1) | 27(50.9) | 0.38 | 0.85 | 24(46.2) | 27(50.9) | 0.38 | 0.92 | 28(53.8) | 25(47.2) | 0.46 | 0.61 | 25(48.1) | 27(50.9) | 0.38 | 0.66 |
| Taste (poor) | 22(42.3) | 35(66) | **0.01** | **0.02** | 20(38.5) | 32(60.4) | 0.14 | 0.36 | 19(36.5) | 31(58.5) | 0.24 | 0.29 | 18(34.6) | 30(56.6) | 0.38 | 0.67 |
| Appetite (poor) | 17(32.7) | 23(43.3) | 0.30 | 0.93 | 16(30.8) | 24(45.3) | 0.18 | 0.81 | 21(40.4) | 20(37.7) | 0.39 | 0.97 | 19(36.5) | 22(41.5) | 0.45 | 0.66 |
| Lethargy (yes) | 20(38.5) | 19(35.8) | 0.61 | 0.87 | 20(38.5) | 22(41.5) | 0.30 | 0.41 | 20(38.5) | 26(49.1) | 0.75 | 0.12 | 19(36.5) | 20(37.3) | 0.24 | 0.25 |
| Chest pain (yes) | 32(61.5) | 22(41.5) | **0.03** | 0.71 | 33(63.5) | 21(39.6) | **0.01** | 0.32 | 25(48.1) | 29(54.7) | 0.31 | 0.39 | 29(55.8) | 25(47.2) | 0.24 | 0.72 |
| Headache (yes) | 30(57.7) | 24(45.3) | 0.14 | 0.55 | 31(59.6) | 23(43.4) | **0.07** | 0.42 | 24(46.2) | 30(56.6) | 0.19 | 0.27 | 29(55.8) | 25(47.2) | 0.24 | 0.37 |
| Vomiting (yes) | 5(9.6) | 9(17.0) | 0.20 | 0.59 | 5(9.6) | 9(17.0) | 0.20 | 0.87 | 5(9.6) | 9(17.0) | 0.20 | 0.10 | 6(11.5) | 8(15.1) | 0.40 | 0.85 |
| Nausea (yes) | 23(44.2) | 22(41.5) | 0.46 | 0.52 | 21(40.4) | 24(45.3) | 0.37 | 0.50 | 20(38.5) | 25.(47.2) | 0.24 | 0.57 | 21(40.4) | 24(45.3) | 0.37 | 0.97 |
| Sore throat (yes) | 17(32.7) | 17(32.1) | 0.55 | 0.59 | 17(32.7) | 17(32.1) | 0.55 | 0.88 | 19(36.5) | 15(28.3) | 0.24 | 0.77 | 19(36.5) | 15(28.3) | 0.24 | 0.30 |
| Confusion (yes) | 16(30.8) | 16(30.2) | 0.55 | 0.31 | 18(31.4) | 14(26.4) | 0.24 | 0.10 | 18(34.6) | 14(26.4) | 0.24 | 0.44 | 19(36.5) | 13(24.5) | 0.13 | 0.51 |
| Fever (yes) | 39(75) | 37(69.8) | 0.35 | 0.71 | 38(73.1) | 38(71.7) | 0.52 | 0.75 | 36(69.2) | 40(75.5) | 0.31 | 0.70 | 37(71.2) | 39(73.6) | 0.47 | 0.70 |
| Chills (yes) | 32(61.5) | 33(62.3) | 0.54 | 0.59 | 31(59.6) | 34(64.2) | 0.39 | 0.96 | 32(61.5) | 33(62.3) | 0.54 | 0.80 | 31(59.6) | 34(64.2) | 0.39 | 0.65 |
| RDS (yes) | 13(25) | 10(18.9) | 030 | 0.29 | 15(28.8) | 8(15.1) | **0.07** | 0.13 | 12(23.1) | 11(20.8) | 0.47 | 0.20 | 14(26.5) | 9(17) | 0.16 | 0.60 |
| Abbreviation: SD, Standard deviation; DH, Duration of hospitalization; RDS, Respiratory distress syndrome; CRP, C-reactive protein; Quantitative variables as means ± SD obtained from the independent t-test.  Categorical variables N (%) obtained from the Chi-square analysis. Significant associations with a *P-value* < 0.05 are bolded. *P-value*^*^ adjusted for age, BMI, energy, physical activity and education level. | | | | | | | | | | | | | | | | |
